# Analyzing the mechanisms that facilitate the subtype-specific assembly of *γ*-aminobutyric acid type A receptors

**DOI:** 10.3389/fnmol.2022.1017404

**Published:** 2022-10-03

**Authors:** Catherine Choi, Joshua L. Smalley, Abigail H. S. Lemons, Qiu Ren, Christopher E. Bope, Jake S. Dengler, Paul A. Davies, Stephen J. Moss

**Affiliations:** ^1^Department of Neuroscience, Tufts University School of Medicine, Boston, MA, United States; ^2^Department of Neuroscience, Physiology and Pharmacology, University College London, London, United Kingdom

**Keywords:** trafficking, subunit composition, phosphorylation, GABA_*A*_ receptors, protein purification

## Abstract

Impaired inhibitory signaling underlies the pathophysiology of many neuropsychiatric and neurodevelopmental disorders including autism spectrum disorders and epilepsy. Neuronal inhibition is regulated by synaptic and extrasynaptic *γ*-aminobutyric acid type A receptors (GABA_*A*_Rs), which mediate phasic and tonic inhibition, respectively. These two GABA_*A*_R subtypes differ in their function, ligand sensitivity, and physiological properties. Importantly, they contain different α subunit isoforms: synaptic GABA_*A*_Rs contain the α1–3 subunits whereas extrasynaptic GABA_*A*_Rs contain the α4–6 subunits. While the subunit composition is critical for the distinct roles of synaptic and extrasynaptic GABA_*A*_R subtypes in inhibition, the molecular mechanism of the subtype-specific assembly has not been elucidated. To address this issue, we purified endogenous α1- and α4-containing GABA_*A*_Rs from adult murine forebrains and examined their subunit composition and interacting proteins using liquid chromatography coupled with tandem mass spectrometry (LC-MS/MS) and quantitative analysis. We found that the α1 and α4 subunits form separate populations of GABA_*A*_Rs and interact with distinct sets of binding proteins. We also discovered that the β3 subunit, which co-purifies with both the α1 and α4 subunits, has different levels of phosphorylation on serines 408 and 409 (S408/9) between the two receptor subtypes. To understand the role S408/9 plays in the assembly of α1- and α4-containing GABA_*A*_Rs, we examined the effects of S408/9A (alanine) knock-in mutation on the subunit composition of the two receptor subtypes using LC-MS/MS and quantitative analysis. We discovered that the S408/9A mutation results in the formation of novel α1α4-containing GABA_*A*_Rs. Moreover, in S408/9A mutants, the plasma membrane expression of the α4 subunit is increased whereas its retention in the endoplasmic reticulum is reduced. These findings suggest that S408/9 play a critical role in determining the subtype-specific assembly of GABA_*A*_Rs, and thus the efficacy of neuronal inhibition.

## Introduction

*γ*-aminobutyric acid type A receptors (GABA_*A*_Rs) are ligand-gated ion channels that mediate neuronal inhibition in the adult brain (Farrant and Nusser, 2005). As the primary regulators of neuronal inhibition, they are also the therapeutic sites of action for benzodiazepines, barbiturates, intravenous anesthetics, and neurosteroids (Rudolph and Möhler, 2006). GABA_*A*_Rs are heteropentamers that can be assembled from the α1–6, β1–3, *γ*1–3, δ, ∈, θ, π, and ρ1–3 subunits (Rudolph and Möhler, 2006). This subunit diversity provides the structural basis for the generation of distinct GABA_*A*_R subtypes endowed with distinct physiological and pharmacological properties. While the identity of the endogenous GABA_*A*_R subtypes that are assembled in neurons remains to be explored, consensus opinion suggests that phasic inhibition is principally mediated by receptor subtypes composed of the α1–3, β1–3, and *γ*2 subunits (Farrant and Nusser, 2005). Tonic inhibition is believed to be mediated by specialized populations of extrasynaptic GABA_*A*_Rs, which are composed of the α4–6 and β2–3 subunits with or without the *γ* or δ subunit (Farrant and Nusser, 2005).

Individual neurons such as dentate gyrus granule cells (DGGCs) or CA1 principal neurons exhibit both robust phasic and tonic currents. Consistent with this, immunohistochemical studies have shown that single neurons can express the α1–5, β1–3, *γ*1–3, and δ subunits (Fritschy and Mohler, 1995; Sperk et al., 1997; Pirker et al., 2000). Significantly, there is accumulating experimental evidence showing that the efficacy of phasic and tonic inhibition can be dynamically modulated by reproductive hormones and neurosteroids (Adams et al., 2015; Modgil et al., 2017, 2019; Mukherjee et al., 2017; Parakala et al., 2019). Moreover, deficits in the “equilibrium” between phasic and tonic inhibition are widely believed to play central roles in the pathophysiology of anxiety, depression, epilepsy, and autism spectrum disorders (Farrant and Nusser, 2005; Benham et al., 2014; Tang et al., 2021).

To date, recombinant expression has been used to understand how hetero-oligomeric GABA_*A*_Rs are constructed from their component subunits. Such experiments have revealed that individual GABA_*A*_R subunits are assembled into heteropentamers in the endoplasmic reticulum (ER) (Jacob et al., 2008). GABA_*A*_Rs that reach ‘conformation maturity’ and form αβ-, αβ*γ*-, or αβδ-containing receptors exit the ER for transport to and insertion into the plasma membrane (PM) (Connolly et al., 1996; Jacob et al., 2008; Vithlani et al., 2011). Unassembled subunits and transport incompetent combinations are targeted for proteasomal degradation (Jacob et al., 2008). Both synaptic and extrasynaptic GABA_*A*_Rs enter the PM at extrasynaptic locations and can access the synaptic space (Thomas et al., 2005; Bogdanov et al., 2006). However, only the receptors that contain the α1–3 subunits are selectively stabilized at synapses due to their affinity for proteins within the postsynaptic scaffold such as gephyrin (Moss and Smart, 2001; Jacob et al., 2008). Thus, the subunit composition of a GABA_*A*_R, and specifically the α subunit isoform, contributes to the receptor’s localization within the PM which is critical for its function. However, how neurons orchestrate the segregation of the α subunit variants into distinct receptor subtypes that mediate phasic and tonic inhibition remains to be defined.

To address this issue, we isolated native GABA_*A*_R subtypes containing the α1 and α4 subunits from the murine forebrain and used quantitative mass spectrometry to examine their subunit composition. We found that the α1 and α4 subunits formed discrete populations of GABA_*A*_Rs that associate with distinct sets of binding proteins, but both contained the β3 subunit. Interestingly, our analysis did not detect the δ subunit co-purifying with the α4 subunit. Additionally, the β3 subunit in α1-containing GABA_*A*_Rs exhibited higher levels of phosphorylation on serines 408 and 409 (S408/9) than the β3 subunit in the α4 subtype. Previous studies have shown that these residues are critical sites for phospho-dependent regulation of GABA_*A*_R membrane trafficking, which impacts the efficacy of GABAergic inhibition (Kittler et al., 2005; Vien et al., 2015). Mutation of S408/9 compromised the segregation of the α1 and α4 subunits into biochemically distinct receptor subtypes. Mechanistically this mutation acted to reduce the ER retention of the α4 subunit and to increase its accumulation in the PM. Thus, our results suggest a critical role for the β3 subunit and its phosphorylation on S408/9 in orchestrating the subtype-specific assembly of GABA_*A*_Rs.

## Results

### Purification and analysis of native GABA_*A*_Rs that are assembled from the α1 subunit

**FIGURE 1:**
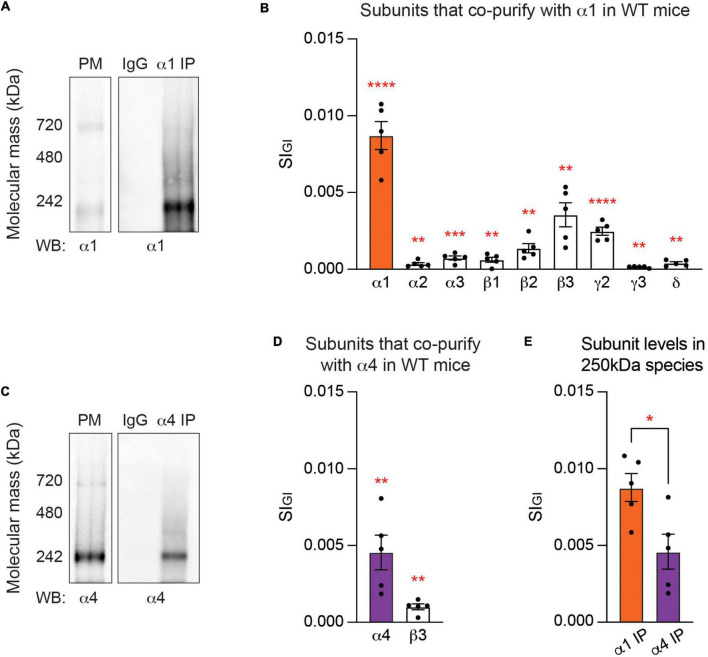
At steady state, the endogenous α1 and α4 subunits are segregated into distinct GABA_*A*_ subtypes. **(A)** Purified plasma membrane fraction (PM), PM exposed to non-immune IgG, and PM exposed to the α1 subunit antibody for purification of GABA_*A*_Rs that contain the α1 subunit (α1 IP) were resolved on Blue Native PAGE (BN-PAGE) gels and probed for the α1 subunit. Major bands at around 250 and 720 kDa, which represent heteropentameric GABA_*A*_Rs in isolation and heteropentameric GABA_*A*_Rs with binding partners, respectively, were observed. **(B)** Gel regions containing the 250 kDa band were subjected to liquid chromatography coupled with tandem mass spectrometry (LC-MS/MS). Analysis of the 250 kDa band with label-free quantification and Welch’s *t*-test revealed that in α1-containing GABA_*A*_Rs, the α1–3, β1–3, *γ*2–3, and δ subunits are significantly enriched when compared to non-immune IgG control. Detected but non-significant subunits are not shown (**p < 0.01, ***p < 0.001, ****p < 0.0001, *n* = 5 replicates). **(C)** Purified PM, PM exposed to non-immune IgG, and PM exposed to the α4 subunit antibody (α4 IP) were resolved on BN-PAGE and probed with the α4 subunit antibody. A band at 250 kDa and a weaker band at 720 kDa were observed with immunoblotting. **(D)** The contents of the 250 kDa band were analyzed with LC-MS/MS. Only the α4 and β3 subunits are significantly enriched in α4-containing GABA_*A*_Rs when compared to non-immune IgG (**p < 0.01, *n* = 5 replicates). **(E)** The amount of target subunit recovery in 250 kDa bands is higher for the α1 subunit than the α4 subunit (*p < 0.05, *n* = 5 replicates).

While it is widely accepted that GABA_*A*_Rs containing the α1 subunit are major mediators of phasic inhibition (Farrant and Nusser, 2005), quantitative analysis of their subunit composition in the neuronal PM has not been reported. To address this issue, adult murine forebrain tissues were collected and homogenized in isotonic, detergent-free buffers and subjected to serial centrifugation for isolation of highly purified PM fractions (Suski et al., 2014; Smalley et al., 2020). Purified PM was solubilized in 0.5% Triton X-100, a detergent that efficiently extracts GABA_*A*_Rs from their native environment without compromising their structure (Chen et al., 2012). To assess the molecular mass of native α1-containing GABA_*A*_Rs in PM, we subjected the purified material to Blue Native PAGE (BN-PAGE) and immunoblotted with the α1 subunit antibody. Two major bands were identified - one at around 250 kDa, which is consistent with the pentameric structure of GABA_*A*_Rs, and the other at around 720 kDa (Figure 1A). Next, the solubilized PM fraction was exposed to a monoclonal antibody against the α1 subunit or non-immune mouse IgG immobilized on magnetic beads and eluted in a buffer containing 2% Tween and 0.01% SDS, a combination sufficient to elute the material from the beads without disturbing stable protein complexes (Antrobus and Borner, 2011). As measured using immunoblotting, high levels of the α1 subunit were retained on the α1 subunit antibody compared to flow through (Supplementary Figure 1A). The eluted material was resolved on BN-PAGE and visualized using immunoblotting. Both 250 and 720 kDa bands were detected in the eluted material with the α1 subunit antibody but not in material purified on IgG control (Figure 1A).

To interrogate the subunit composition of GABA_*A*_Rs containing the α1 subunit, the gel area containing the lower molecular weight species at around 250 kDa was excised and its contents were analyzed with liquid chromatography coupled with tandem mass spectrometry (LC-MS/MS). The LC-MS/MS data was analyzed with label-free, globally normalized quantification. Briefly, the spectral index of each protein was normalized to all proteins detected in the sample (SI_*GI*_), which allowed quantitative comparison of protein amounts within and between biologically distinct data sets (Griffin et al., 2010; Smalley et al., 2020). The SI_*GI*_ values of proteins detected in a purification were compared to those detected in non-immune IgG control, and Welch’s *t*-test was performed to identify significantly enriched subunits. This approach revealed that the 250 kDa species was highly enriched in GABA_*A*_R subunits (Supplementary Table 1). Specifically, we found that the α1–3, β1–3, *γ*2–3, and δ subunits are significantly enriched in major populations of α1-containing GABA_*A*_Rs, compared to the IgG control (Figure 1B and Supplementary Table 1A). The most abundant subunits were the α1 subunit (SI_*GI*_ of 0.0087 ± 0.0009, *p* < 0.0001, *n* = 5, Welch’s *t*-test), followed by the β3 (0.0035 ± 0.0008, *p* = 0.0026, *n* = 5, Welch’s *t*-test), *γ*2 (0.0024 ± 0.0003, *p* < 0.0001, *n* = 5, Welch’s *t*-test), β2 (0.0014 ± 0.0003, *p* = 0.0027, *n* = 5, Welch’s *t*-test), α3 (0.0007 ± 0.0001, *p* = 0.0009, *n* = 5, Welch’s *t*-test), β1 (0.0006 ± 0.0002, *p* = 0.0069, *n* = 5, Welch’s *t*-test), δ (0.0004 ± 0.00009, *p* = 0.0022, *n* = 5, Welch’s *t*-test), α2 (0.0003 ± 0.00009, *p* = 0.0094, *n* = 5, Welch’s *t*-test), and *γ*3 (0.0001 ± 0.00003, *p* = 0.0050, *n* = 5, Welch’s *t*-test) subunits (Figure 1B and Supplementary Table 1A). Based on the relative abundance, α1β3*γ*2 is likely to be the most common subunit composition of α1-containing GABA_*A*_Rs. The α4 (0.0002 ± 0.00008, *p* = 0.1194, *n* = 5, Welch’s *t*-test), α5 (0.0004 ± 0.0002, *p* = 0.0723, *n* = 5, Welch’s *t*-test), and *γ*1 (0.00003 ± 0.00003, *p* = 0.2035, *n* = 5, Welch’s *t*-test) subunits were detected but not significantly enriched when compared to IgG control.

### Purification and analysis of native GABA_*A*_Rs that are assembled from the α4 subunit

We also analyzed the subunit composition of GABA_*A*_Rs that contain the α4 subunit, which are the principal mediators of tonic current in the adult forebrain (Chandra et al., 2006). To quantitatively analyze the subunit composition of α4-containing GABA_*A*_Rs in the neuronal PM, we first subjected purified PM fractions from murine forebrain tissues to BN-PAGE and immunoblotted with the α4 subunit antibody. A major band at around 250 kDa and a weaker band at around 720 kDa were observed (Figure 1C). The PM fraction was then subjected to immunoprecipitation with an immobilized monoclonal antibody against the α4 subunit or non-immune mouse IgG and eluted under non-denaturing conditions as described above. The effectiveness of immunoprecipitation with the α4 subunit antibody was verified via immunoblotting (Supplementary Figure 1B). The eluted material was resolved by BN-PAGE and visualized using immunoblotting, which revealed the 250 kDa band in the eluted material purified with the α4 subunit antibody (Figure 1C). No immunoreactivity was observed with IgG control (Figure 1C).

LC-MS/MS was performed on the major band at 250 kDa and the data were analyzed with label-free normalized quantification and Welch’s *t*-test. We found that only the α4 (0.0046 ± 0.0011, *p* = 0.0049, *n* = 5, Welch’s *t*-test) and β3 (0.0010 ± 0.0002, *p* = 0.0013, *n* = 5, Welch’s *t*-test) subunits are significantly enriched in major populations of α4-containing GABA_*A*_Rs, when compared to IgG control (Figure 1D and Supplementary Table 1B). The α5, *γ*1, and *γ*3 subunits were not detected at all, and others – the α1 (0.0001 ± 0.00007, *p* = 0.0967, *n* = 5, Welch’s *t*-test), α2 (0.00003 ± 0.00003, *p* = 0.4858, *n* = 5, Welch’s *t*-test), α3 (0.0002 ± 0.0002, *p* = 0.2035, *n* = 5, Welch’s *t*-test), α6 (0.0002 ± 0.0002, *p* = 0.0994, *n* = 5, Welch’s *t*-test), β1 (0.00003 ± 0.00002, *p* = 0.0973, *n* = 5, Welch’s *t*-test), β2 (0.0001 ± 0.00008, *p* = 0.1226, *n* = 5, Welch’s *t*-test), *γ*2 (0.000006 ± 0.00006, *p* = 0.2035, *n* = 5, Welch’s *t*-test), and δ (0.00008 ± 0.00005, *p* = 0.0983, *n* = 5, Welch’s *t*-test) subunits – were detected in low amounts but not significantly enriched when compared to IgG control. While the α1 and α4 subunits are mutually exclusive, the β3 subunit is significantly enriched in both α1- and α4-containing populations of GABA_*A*_Rs (Figures 1B,D and Supplementary Tables 1A,B).

A significant limitation in comparing data sets between α1- and α4-containing GABA_*A*_Rs is controlling for the recovery of the target receptor subtype between purifications. To control for this variable, we directly compared the amount of each target α subunit isolated in the respective purifications. We found that there is an approximately twofold higher level of recovery of the α1 subunit compared to the α4 subunit (Figure 1E; α1 IP: 0.0087 ± 0.0009, α4 IP: 0.0046 ± 0.0011, *p* = 0.0211, *n* = 5, unpaired *t*-test). The lower recovery of the α4 subunit is likely to reflect that the α1 subunit is expressed at about 15-fold higher levels than the α4 subunit in the brain, as quantified by mass spectrometry (Chen et al., 2012). Collectively, our experiments strongly suggest that neurons have the capacity to segregate the α1 and α4 subunits into distinct receptor subtypes with distinct subunit composition.

### Analysis of the proteins that co-purify with α1-containing GABA_*A*_Rs

**FIGURE 2:**
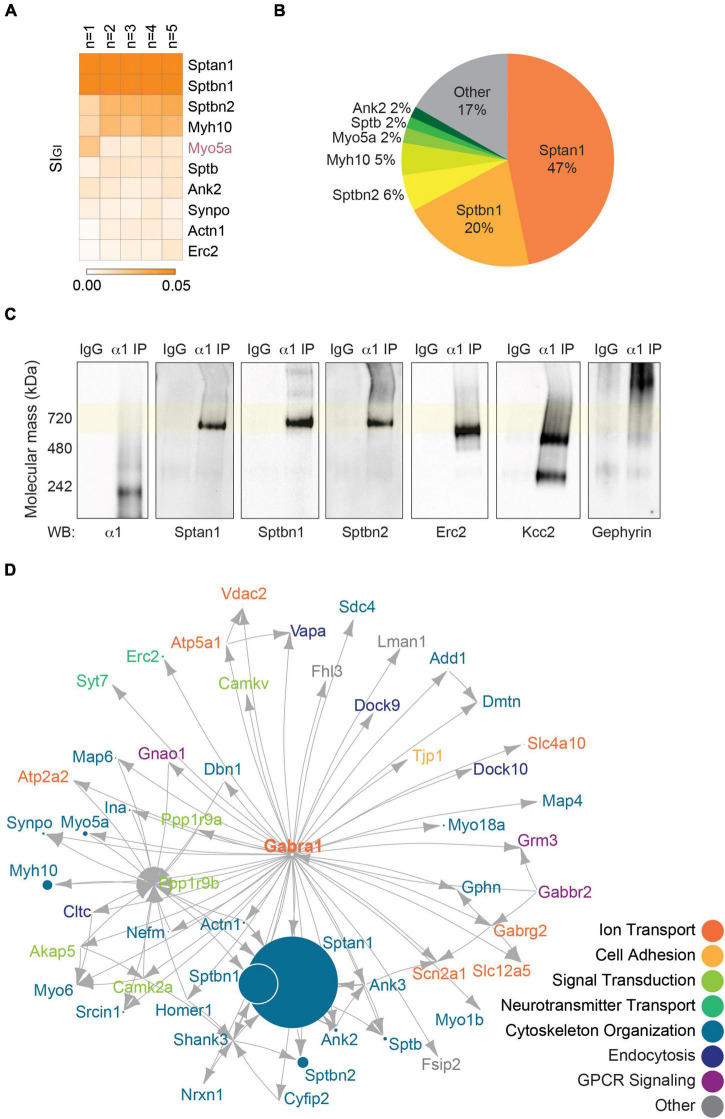
The endogenous α1 subunit interacts with inhibitory scaffolding proteins and cytoskeletal proteins. **(A)** The 720 kDa band of α1-containing GABA_*A*_Rs purified from PM was subjected to LC-MS/MS. Label-free normalized quantification and Welch’s *t*-test against non-immune control IgG revealed 93 significantly enriched binding proteins of the α1 subunit. The heatmap shows the top 10 binding proteins of α1-containing GABA_*A*_Rs by abundance (SI_GI_ value) across all replicates. The most abundant proteins detected with the α1 subunit are Sptan1, Sptbn1, and Sptbn2 (*n* = 5 replicates). **(B)** Pie chart shows the relative abundance of the proteins that are significantly enriched with α1-containing GABA_*A*_Rs. Sptan1, Sptbn1, and Sptbn2 together constitute more than 70% of the total amount of proteins. Proteins that make up less than 2% of the total amount of proteins (86 proteins) were grouped together as ‘Other.’ **(C)** Immunopurified α1-containing GABA_*A*_Rs were resolved on BN-PAGE and probed for the α1 subunit, high-abundance proteins (Sptan1, Sptbn1, and Sptbn2), and low-abundance proteins (Erc2, Kcc2, and gephyrin). Sptan1, Sptbn1, and Sptbn2 are observed at around 720 kDa with α1-containing GABA_*A*_Rs. Erc2 and Kcc2 are present slightly below 720 kDa and gephyrin is observed higher than 720 kDa. The immunoreactivity of these proteins is specific to purified α1-containing GABA_*A*_Rs. **(D)** The network analysis shows known inhibitory scaffolding proteins such as Actn1, Cyfip2, and Gphn (gephyrin), and a subnetwork of spectrins (Sptan1, Sptbn1, Sptbn2, and Sptb) and ankyrins (Ank2 and Ank3). GO analysis reveals that most proteins are involved in cytoskeletal organization, ion transport, or signal transduction.

In addition to GABA_*A*_R subunits that co-purify with the α1 subunit, we examined which proteins associate with α1-containing GABA_*A*_Rs. To do so, we used LC-MS/MS and quantitative analysis to reveal the contents of the gel region containing the 720 kDa bands, which were evident in purifications of α1-containing GABA_*A*_Rs. Welch’s *t*-test was performed against non-immune mouse IgG to identify significantly enriched proteins, and only those that were detected in all replicates were included for subsequent analysis. For the α1 subunit, 93 proteins were identified as significantly enriched proteins (Supplementary Table 2A). Across all replicates, the most abundantly found proteins are Sptan1 (0.2476 ± 0.0426, *p* = 0.0008, *n* = 5, Welch’s *t*-test), Sptbn1 (0.1078 ± 0.0165, *p* = 0.0005, *n* = 5, Welch’s *t*-test), and Sptbn2 (0.0292 ± 0.0031, *p* < 0.0001, *n* = 5, Welch’s *t*-test), which are different isoforms of the spectrin family (Figure 2A and Supplementary Table 2A). Together these isoforms of spectrin constitute over 70% of all the proteins co-purified with the α1 subunit – 47% Sptan1, 20% Sptbn1, and 6% Sptbn2 (Figure 2B). Each of the remaining 90 proteins makes up 5% or less of the total amount of proteins detected with α1-containing GABA_*A*_Rs (Figure 2B). These suggest that the detection of the high molecular weight species of α1-containing GABA_*A*_Rs at around 720 kDa is mainly driven by the presence of spectrin isoforms. Other notable proteins that are significantly enriched with the α1 subunit are known inhibitory scaffolding proteins such as Cyfip2 (0.0008 ± 0.0002, *p* = 0.0074, *n* = 5, Welch’s *t*-test), and Gphn (gephyrin; 0.0005 ± 0.0001, *p* = 0.0017, *n* = 5, Welch’s *t*-test) (Kneussel et al., 1999; Davenport et al., 2019) (Supplementary Table 2A).

To confirm the association of the proteins identified by LC-MS/MS with the α1 subunit, we resolved purified α1-containing GABA_*A*_Rs on BN-PAGE and immunoblotted with antibodies against some of the proteins detected in high- and low-abundance. For high-abundance proteins, we probed for Sptan1, Sptbn1, and Sptbn2 and observed their immunoreactivity at around 720 kDa (Figure 2C). We also assessed the presence of low-abundance interactors – Erc2, Kcc2 (Slc12a5), and gephyrin. For Erc2 and Kcc2, their immunoreactivity was observed slightly below 720 kDa, with Kcc2 showing an additional band between 242 and 480 kDa (Figure 2C). Gephyrin was observed higher than 720 kDa, at approximately 1,048 kDa (Figure 2C). For these high- and low-abundance interactors of the α1 subunit, the immunoreactivity was specific to purified α1-containing GABA_*A*_Rs (Figure 2C), which provided confidence in the veracity of our approach.

Next, we performed the network analysis on the significantly enriched proteins of the α1 subunit to gain insights into the known relationships between the proteins. For the analysis, we included proteins that are (1) significantly enriched, (2) detected in all replicates, and (3) have average SI_*GI*_ values greater than 0.0005. We used STRINGdb database to impute known interactions between proteins (Szklarczyk et al., 2019) and scaled each node to the average SI_*GI*_ value of the protein. Lastly, we overlaid the highest scoring Gene Ontology (GO) Biological Process term for each protein to provide information on the protein function (Szklarczyk et al., 2019). The network of the α1 subunit shows interactions between the spectrin isoforms (Sptan1, Sptbn1, Sptbn2, and Sptb) and ankyrin isoforms (Ank2 and Ank3), as well as interactions between gephyrin (Gphn) and Kcc2 (Slc12a5) (Figure 2D). Many of the associated proteins of the α1 subunit are involved in cytoskeleton organization, ion transport, or signal transduction (Figure 2D). These results show that the α1 subunit interacts with scaffolding and cytoskeletal proteins that stabilize α1-containing GABA_*A*_Rs in the PM.

### Analysis of the proteins that co-purify with α4-containing GABA_*A*_Rs

**FIGURE 3:**
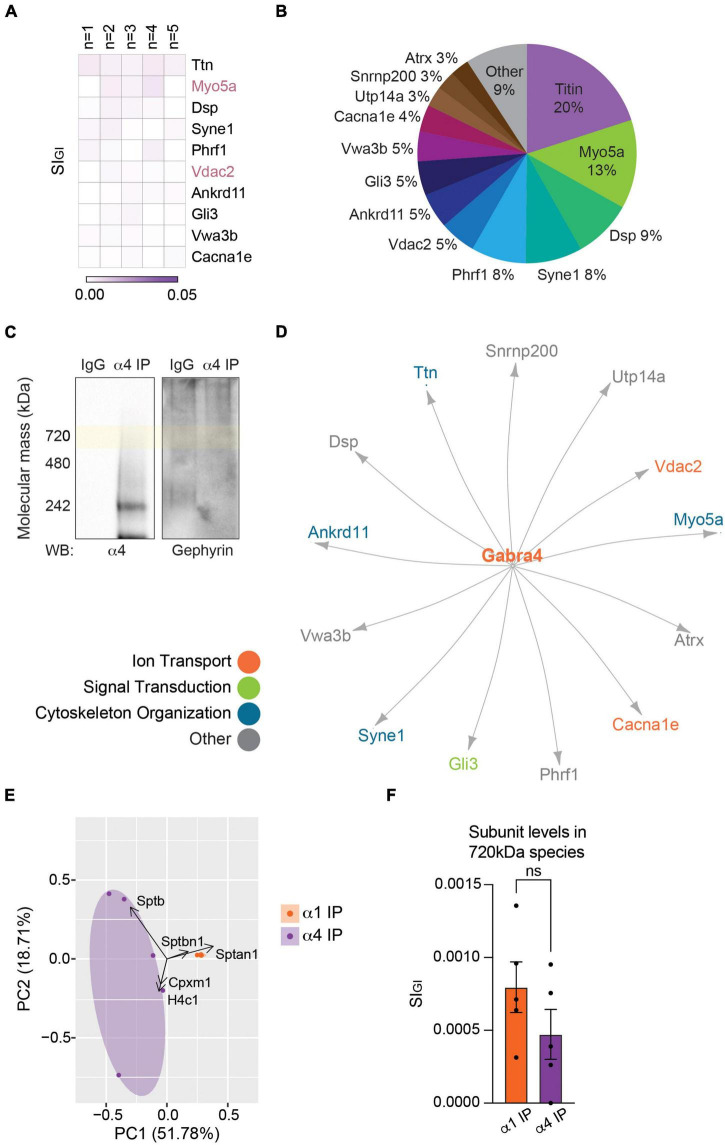
The endogenous α4 subunit does not interact with inhibitory scaffolding proteins. **(A)** Analysis of the 720 kDa band of α4-containing GABA_*A*_Rs with LC-MS/MS and quantitative analysis identified 19 binding proteins. The heatmap shows the top 10 binding proteins by abundance, which include Ttn (titin), Myo5a, and Dsp (desmoplakin). Myo5a and Vdac2 were detected with both the α1 and α4 subtypes (*n* = 5 replicates). **(B)** The relative abundance of binding proteins of the α4 subunit is illustrated as a pie chart. Besides 20% by titin and 13% by Myo5a, most proteins constitute less than 10% of the total amount of proteins detected with α4-containing GABA_*A*_Rs. Proteins that make up less than 2% each (6 proteins) were grouped together as ‘Other.’ **(C)** BN-PAGE blots of purified α4-containing GABA_*A*_Rs show the absence of gephyrin in the α4 subtype. **(D)** The network analysis reveals no known interactions among the proteins detected with the α4 subunit. **(E)** PCA plot of the significantly enriched binding proteins of the α1 and α4 subunits show high reproducibility of datasets between replicates. It also highlights the difference in the binding proteins of the two receptor subtypes. **(F)** Target subunit recovery in 720 kDa bands between subtype purifications is comparable (ns ≥ 0.05, *n* = 5 replicates).

We also analyzed the proteins associated with the α4 subunit as described above. The α4 subunit has 19 significantly enriched proteins, none of which are regarded as inhibitory scaffolding proteins (Supplementary Table 2B). Proteins that interact with the α4 subunit in the order of abundance are Ttn (titin; 0.0039 ± 0.0004, *p* = 0.0464, *n* = 5, Welch’s *t*-test), Myo5a (0.0026 ± 0.0010, *p* = 0.0416, *n* = 5, Welch’s *t*-test), and Dsp (desmoplakin; 0.0017 ± 0.0005, *p* = 0.0271, *n* = 5, Welch’s *t*-test) (Figure 3A and Supplementary Table 2B). Only two proteins were detected with both α1- and α4-containing GABA_*A*_Rs: Myo5a and Vdac2 (Figures 2A, 3A). Unlike in α1-containing GABA_*A*_Rs, there is not a single protein or a family of proteins that dominates the makeup of the proteins purified with α4-containing GABA_*A*_Rs – 20% titin, 13% Myo5a, 9% desmoplakin, followed by 8% or less of each of the remaining 16 proteins (Figure 3B). The inhibitory scaffolding protein gephyrin was not detected with the α4 subunit. Consistent with this, when purified α4-containing GABA_*A*_Rs were resolved on BN-PAGE and immunoblotted with the gephyrin antibody, no immunoreactivity was observed (Figure 3C). The network analysis revealed that there are no known interactions among the proteins detected with the α4 subunit (Figure 3D). These results suggest that the α4 subunit does not interact with components of the sub-synaptic cytoskeleton.

### Comparing the multiprotein complexes of α1- and α4-containing GABA_*A*_Rs

To visualize the similarity or difference among the associated proteins of α1- and α4-containing GABA_*A*_Rs and to assess the reproducibility of our purifications, we performed the principal component analysis (PCA). The replicates of the α1 subunit were tightly grouped together, suggesting high reproducibility of the α1 subunit purifications (Figure 3E). It also suggests that there is a core group of proteins that is commonly identified with the α1 subunit across all replicates. The replicates of the α4 subunit, on the other hand, were more spread out on the PCA plot (Figure 3E), suggesting the lack of common components across all replicates. Importantly, the two types of purifications were clearly separated (Figure 3E), further suggesting that the α1 and α4 subunits associate with distinct sets of binding proteins. The first principal component (PC1) accounted for 51.78% of the variance and was positively correlated with Sptan1 and Sptbn1, the two most abundantly detected proteins of the α1 subunit (Figure 2). The second principal component (PC2) accounted for 18.71% of the variance. Together, our findings show that the α1 and α4 subunits form separate receptor populations with distinct subunit composition and binding proteins.

Finally, we compared the amount of target subunit detected in the 720 kDa bands between purifications of the two GABA_*A*_R populations. We found that there was no significant difference between the amounts of the α1 and α4 subunits in each purification (Figure 3F; α1 IP: 0.0008 ± 0.0002, α4 IP: 0.0005 ± 0.0002, *p* = 0.2205, *n* = 5, unpaired *t*-test). This suggests that the input amounts of α1- and α4-containing GABA_*A*_Rs in our experiments were comparable.

### Examining the levels of the β3 subunit phosphorylation in GABA_*A*_Rs assembled from the α1 and α4 subunits

**FIGURE 4:**
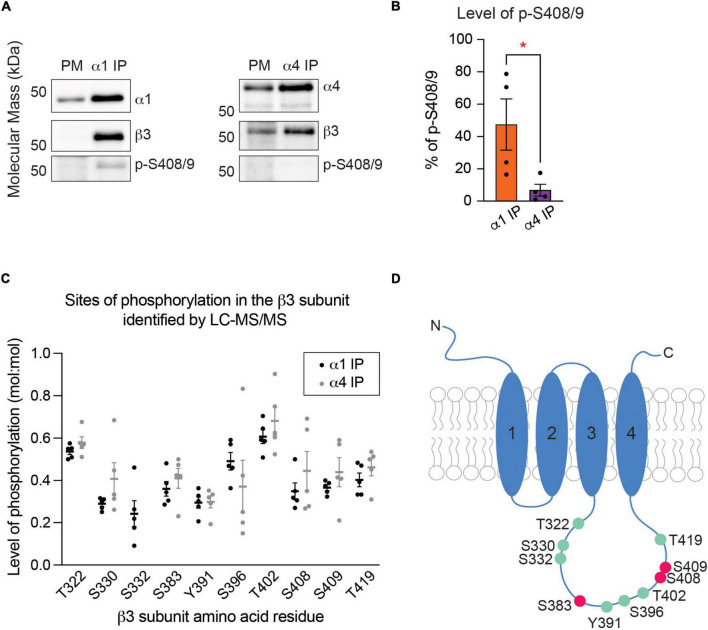
The β3 subunit is more highly phosphorylated at S408 and S409 in α1-containing GABA_*A*_Rs than in the α4 subtype. **(A)** Immunoblots of purified α1- and α4-containing GABA_*A*_Rs were resolved on SDS-PAGE and probed for the target subunit (α1 or α4 subunit), β3 subunit, and phosphorylated S408/9 (p-S408/9). **(B)** The percentage of p-S408/9 in respect to total β3 subunit is significantly higher in the α1 subtype than in the α4 subtype (*p < 0.05, *n* = 4 replicates). **(C)** Spectra searches were performed on LC-MS/MS data obtained from purified α1- and α4-containing GABA_*A*_Rs to identify phosphorylated serine (S), threonine (T), or tyrosine (Y) residues within the β3 subunit. Three known (S383, S408, and S409) and seven novel (T322, S330, S332, Y391, S396, T402, and T419) phosphorylation sites were detected. S332 was detected in the α1 subtype only (*n* = 5 replicates). **(D)** Illustration of a GABA_*A*_R β3 subunit with phosphorylation sites detected by LC-MS/MS is shown. Known and novel sites are in pink and green, respectively.

The β3 subunit is a common component of both α1- and α4-containing GABA_*A*_Rs (Figures 1B,D and Supplementary Tables 1A,B). It has been reported that simultaneous phosphorylation of this subunit on both serine 408 and serine 409 (S408/9) acts to regulate the membrane trafficking of GABA_*A*_Rs by decreasing their binding to adaptins, which are critical for the retrograde protein transport in the endocytic pathway (Kittler et al., 2005; Vien et al., 2015). To examine whether phosphorylation of S408/9 plays a role in the segregation of the α1 and α4 subunits into distinct receptor subtypes, we purified the two GABA_*A*_R populations from the PM and measured the ratio of phosphorylated S408/9 (p-S408/9) to total β3 subunit using a phospho-specific antibody that only recognizes the β3 subunit when both S408 and S409 are phosphorylated (Brandon et al., 2002b,^2003^; Jovanovic et al., 2004; Saliba et al., 2012; Vien et al., 2015; Parakala et al., 2019). This approach revealed that the percentage of p-S408/9 was significantly higher in α1-containing GABA_*A*_Rs than in the α4 subtype (Figures 4A,B; α1 IP: 47.48 ± 15.85%, α4 IP: 6.76 ± 3.74%, *p* = 0.0465, *n* = 4, unpaired *t*-test).

To evaluate whether there are other phosphorylation sites within the β3 subunit, we compared global β3 subunit phosphorylation between the two receptor subtypes using LC-MS/MS. Spectral searches were performed to identify phosphorylated serine (S), threonine (T), or tyrosine (Y) residues on the intracellular loop domain of the β3 subunit, which is the site of post-translational modifications that regulate receptor trafficking and PM expression of GABA_*A*_Rs (Moss et al., 1992; Brandon et al., 2002a; Nymann-Andersen et al., 2002; O’Toole and Jenkins, 2011). The levels of phosphorylation on these residues were then estimated by comparing the number of phosphorylated and dephosphorylated peptides. This approach is valid as our purifications do not employ a phospho-enrichment step. In receptors that contain the α1 subunit, in addition to peptides phosphorylated on S408 or S409, LC-MS/MS analysis detected phosphorylated S383 (Figure 4C), which is a previously identified phosphorylation site that is modulated by calcium/calmodulin-dependent protein kinase (Saliba et al., 2012). Seven additional novel sites of phosphorylation – T322, S330, S332, Y391, S396, T402, and T419 – were also evident with mean phosphopeptide ratios of 0.2 – 0.7 (Figure 4C). Similar levels of phosphorylation were found at these residues in α4-containing GABA_*A*_Rs, except at S332 where phosphorylation was not detected (Figure 4C). These results demonstrate that α1β3- and α4β3-containing GABA_*A*_Rs are phosphorylated at multiple sites within the intracellular loop domain of the β3 subunit (Figure 4D).

### Mutation of S408/9 does not change the total expression levels of the α1 or α4 subunit

**FIGURE 5:**
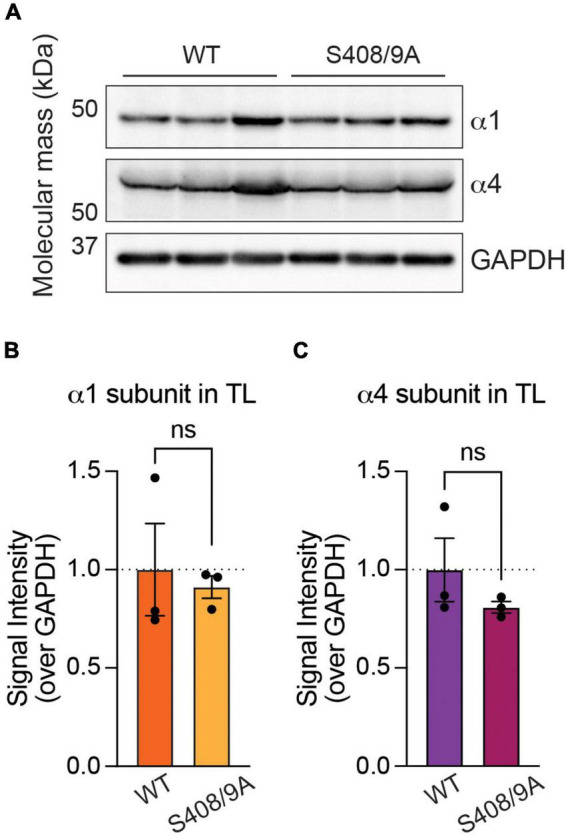
Mutation of S408/9 does not change the total expression level of the α1 or α4 subunit. **(A)** Immunoblots of forebrain tissues from WT and S408/9A mice were probed for the α1 subunit, α4 subunit, and GAPDH. **(B)** Signal intensity of the α1 subunit was normalized to that of GAPDH. There is no significant change in the total level of the α1 subunit in the forebrain between WT and S408/9A mice (ns ≥ 0.05, *n* = 3). **(C)** The S408/9A mutation does not affect the total level of the α4 subunit in the forebrain (ns ≥ 0.05, *n* = 3).

To determine whether S408/9 contribute to the subtype-specific assembly of GABA_*A*_Rs, we utilized mice in which S408/9 have been mutated to alanines (S408/9A). These animals are viable but exhibit increased seizure sensitivity, increased autism-like behaviors, and deficits in GABAergic inhibition (Vien et al., 2015). Thus, we investigated whether the ability of neurons to segregate the α1 and α4 subunits into distinct receptor subtypes is disrupted in animals carrying the S408/9A mutation. We first examined if the mutation alters the total expression levels of the α1 or α4 subunit in the forebrain. Levels of the α1 and α4 subunits were measured in the forebrain tissues collected from WT and S408/9A animals and normalized to GAPDH as the loading control. We found that there was no change in the level of the α1 (S408/9A: 0.9113 ± 0.0570, *p* = 0.7312, *n* = 3, unpaired *t*-test) or α4 subunit (S408/9A: 0.8097 ± 0.0293, *p* = 0.3114, *n* = 3, unpaired *t*-test) (Figure 5).

### Mutation of S408/9 leads to the formation of α1α4-containing GABA_*A*_Rs

**FIGURE 6:**
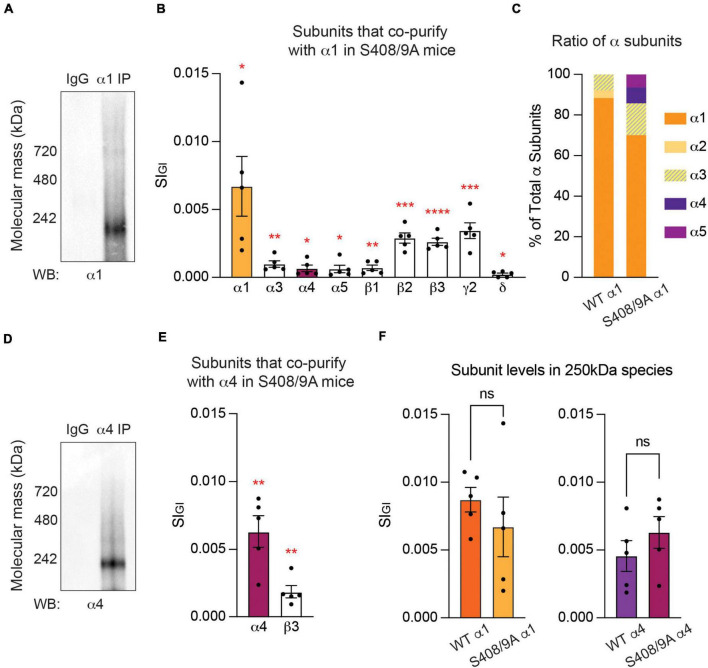
Mutation of S408/9 leads to the formation of α1α4-containing GABA_*A*_Rs. **(A)** Purified PM from S408/9A mice were exposed to non-immune control IgG or the α1 subunit antibody and probed for the α1 subunit. Major bands at 250 and 720 kDa were observed. **(B)** Quantitative analysis of the contents of the 250 kDa band revealed that the α4 and α5 subunits are significantly enriched in α1-containing GABA_*A*_Rs in S408/9A mice, along with the α1, α3, β1–3, *γ*2, and δ subunits (*p < 0.05, **p < 0.01, ***p < 0.001, ****p < 0.0001, *n* = 5 replicates). **(C)** Contribution of each α subunit variant to the total amount of α subunits is compared between α1-containing GABA_*A*_Rs from WT and S408/9A mice. With the S408/9A mutation, the percentages of the α1 and α2 subunits decrease whereas those of the α3, α4, and α5 subunits increase. **(D)** The α4 subunit antibody recognized a major band at 250 kDa in purified α4-containing GABA_*A*_Rs from S408/9A mice. **(E)** In α4-containing GABA_*A*_Rs from S408/9A mice, only the α4 and β3 subunits are significantly enriched (**p < 0.01, *n* = 5 replicates). **(F)** The S408/9A mutation does not affect the efficacy of the purification of either α1- or α4-containing GABA_*A*_Rs (ns ≥ 0.05, *n* = 5 replicates).

Next, we examined the effects of the S408/9A mutation on the subunit composition of receptors assembled from the α1 subunit. To do so, α1-containing GABA_*A*_Rs were purified from PM fractions collected from S408/9A mice, subjected to BN-PAGE, and visualized with the α1 subunit antibody. Consistent with our experiments in WT, bands at around 250 and 720 kDa were observed (Figure 6A). The major band at around 250 kDa was then examined using LC-MS/MS and label-free quantitative analysis as detailed above. In the α1 subtype, the α1, α3–5, β1–3, *γ*2, and δ subunits are significantly enriched when compared to non-immune IgG control (Figure 6B and Supplementary Table 1C). The most abundantly detected subunit was the α1 subunit (0.0067 ± 0.0022, *p* = 0.0155, *n* = 5, Welch’s *t*-test), followed by the *γ*2 (0.0034 ± 0.0006, *p* = 0.0006, *n* = 5, Welch’s *t*-test), β2 (0.0029 ± 0.0004, *p* = 0.0001, *n* = 5, Welch’s *t*-test), β3 (0.0026 ± 0.0003, *p* < 0.0001, *n* = 5, Welch’s *t*-test), α3 (0.0010 ± 0.0002, *p* = 0.0038, *n* = 5, Welch’s *t*-test), β1 (0.0007 ± 0.0002, *p* = 0.0081, *n* = 5, Welch’s *t*-test), α4 (0.0007 ± 0.0002, *p* = 0.0352, *n* = 5, Welch’s *t*-test), α5 (0.0006 ± 0.0003, *p* = 0.0430, *n* = 5, Welch’s *t*-test), and δ (0.0002 ± 0.00009, *p* = 0.0367, *n* = 5, Welch’s *t*-test) subunits (Figure 6B and Supplementary Table 1C). Subunits that were detected but not found to be significant are the α2 (0.0004 ± 0.0002, *p* = 0.0733, *n* = 5, Welch’s *t*-test), *γ*1 (0.000002 ± 0.000002, *p* = 0.2035, *n* = 5, Welch’s *t*-test), and *γ*3 (0.0001 ± 0.000007, *p* = 0.0793, *n* = 5, Welch’s *t*-test) subunits. From this, the most common composition of α1-containing GABA_*A*_Rs in S408/9A mice can be estimated to be α1β2/3*γ*2.

In WT, the α1–3 subunits are the only α subunit isoforms that are significantly enriched in α1-containing GABA_*A*_Rs (Figure 1B and Supplementary Table 1A). In S408/9A mice, however, in addition to the α1 and α3 subunits, the α4 and α5 subunits are significantly enriched in α1-containing GABA_*A*_Rs (Figure 6B and Supplementary Table 1C). To understand how the presence of these additional α subunit variants affects the stoichiometry of total α subunit isoforms, we assessed the contribution of each α subunit isoform to the total amount of α subunits detected in our purifications from WT and S408/9A animals. In WT, the α1 subunit makes up 88.28% of all α subunits, followed by 3.82% of the α2 subunit and 7.90% of the α3 subunit (Figure 6C). With the S408/9A mutation, however, the contribution of the α1 subunit to the total α subunits decreases to 70.05%, while that of the α3 subunit increases to 15.62% (Figure 6C). The α4 and α5 subunits constitute 7.81% and 6.52% of all α subunits detected, respectively (Figure 6C). Considering that the conventional stoichiometry of a GABA_*A*_R is 2α:2β:1 extra subunit (Wisden et al., 1992; Baumann et al., 2003), the presence of the α4 and α5 subunits in α1-containing GABA_*A*_Rs implies significant changes to the receptor population in S408/9A animals. Taken together, our experimental results demonstrate that receptors that contain both the α1 and α4 subunits are formed in S408/9A mice, suggesting a critical role of S408/9 in the segregation of the two α subunits into distinct receptor subtypes.

We also examined the contents of the 720 kDa band detected with the α1 subunit antibody with LC-MS/MS and quantitative analysis to reveal the significantly enriched proteins of the α1 subunit in S408/9A mice. Compared to 93 in WT, only 38 proteins were significantly enriched with α1-containing GABA_*A*_Rs in S408/9A mice (Supplementary Table 2C). The most abundantly found proteins with the α1 subunit in S408/9A mice are still Sptan1, Sptbn1, and Sptbn2 (Supplementary Figures 2A,B and Supplementary Table 2C). In addition, gephyrin was detected with the α1 subunit (Supplementary Table 2C). Lastly, the mutation did not alter the efficacy of the purification of α1-containing GABA_*A*_Rs (Supplementary Figure 2C). Together, these suggest that the S408/9A mutation does not alter the high-abundance binding partners of α1-containing GABA_*A*_Rs or disrupt binding to core cytoskeletal proteins that regulate the accumulation of this receptor subtype at synapses.

### The subunit composition of α4-containing GABA_*A*_Rs is not affected by the S408/9A mutation

We also analyzed the subunit composition of α4-containing GABA_*A*_Rs in S408/9A mice. When purified GABA_*A*_Rs that contain the α4 subunit from S408/9A mice were resolved on BN-PAGE and immunoblotted with the α4 subunit antibody, the major band of 250 kDa was observed (Figure 6D). We then employed LC-MS/MS to reveal the contents of the 250 kDa band. Among the GABA_*A*_R subunits detected, only the α4 (0.0063 ± 0.0012, *p* = 0.0011, *n* = 5, Welch’s *t*-test) and β3 (0.0019 ± 0.0005, *p* = 0.0045, *n* = 5, Welch’s *t*-test) subunits were found to be significantly enriched when compared to IgG control (Figure 6E and Supplementary Table 1D). The α1 (0.0005 ± 0.0004, *p* = 0.1351, *n* = 5, Welch’s *t*-test), α3 (0.00008 ± 0.00008, *p* = 0.2035, *n* = 5, Welch’s *t*-test), β1 (0.00003 ± 0.00003, *p* = 0.2035, *n* = 5, Welch’s *t*-test), δ (0.0001 ± 0.0001, *p* = 0.1319, *n* = 5, Welch’s *t*-test), and *γ*2 (0.00001 ± 0.000008, *p* = 0.1172, *n* = 5, Welch’s *t*-test) subunits were detected but not significantly enriched, and the α2, α5–6, β2, *γ*1, and *γ*3 subunits were not detected at all.

To ensure that the S408/9A mutation does not interfere with the efficacy of the purification of GABA_*A*_Rs that contain either the α1 or α4 subunit, we compared the target subunit recovery in each purification between the two genotypes. We found that there is no significant difference in the amount of the α1 (WT: 0.0087 ± 0.0009, S408/9A: 0.0067 ± 0.0022, *p* = 0.4237, *n* = 5, unpaired *t*-test) or α4 (WT: 0.0046 ± 0.0011, S408/9A: 0.0063 ± 0.0012, *p* = 0.3158, *n* = 5, unpaired *t*-test) subunit detected in the 250 kDa band between WT and S408/9A animals (Figure 6F). Collectively, these results suggest that the S408/9A mutation does not alter the subunit composition of α4-containing GABA_*A*_Rs.

Analysis of the 720 kDa band revealed that α4-containing GABA_*A*_Rs in S408/9A mice interact with only 9 proteins, none of which were known inhibitory scaffolding proteins (Supplementary Figures 2D,E and Supplementary Table 2D). In addition, there was no overlap between binding proteins of the α4 subtype between WT and S408/9A mice (Figure 3, Supplementary Figures 2D,E, and Supplementary Tables 2B,D). The mutation had no effect on the efficacy of α4-containing GABA_*A*_R purification (Supplementary Figure 2F). Together, these results suggest the lack of proteins that specifically anchor the α4 subunit in the membrane.

### The S408/9A mutation does not induce gross changes in the basal phosphorylation of the β3 subunit

**FIGURE 7:**
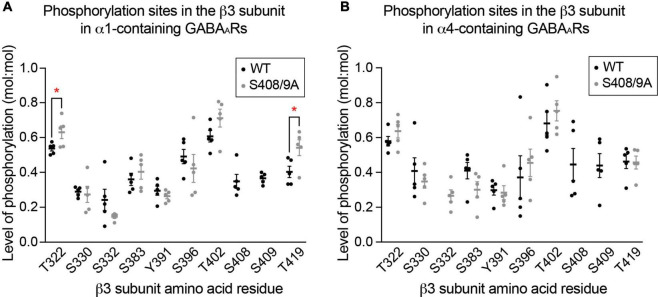
The S408/9A mutation does not lead to gross changes in global β3 subunit phosphorylation. **(A)** Phosphorylated residues within the β3 subunit of α1-containing GABA_*A*_Rs are compared between WT and S408/9A mice. The mutation does not induce aberrant phosphorylation on the β3 subunit, as indicated by the detection of the same phosphorylation sites in WT and S408/9A mice. Two sites – T322 and T419 – are more highly phosphorylated in S408/9A than in WT mice (*p < 0.05, *n* = 5 replicates). **(B)** Phosphorylated residues within the β3 subunit of α4-containing GABA_*A*_Rs from WT and S408/9A mice are shown. Phosphorylated S332 was only detected in the α4 subtype from S408/9A mice (*n* = 5 replicates).

Next, we assessed if mutation of S408/9 induced aberrant phosphorylation on other residues within the β3 subunit in either receptor subtype using LC-MS/MS. We found that in α1-containing GABA_*A*_Rs, the same phosphorylation sites that are present in WT were also detected in S408/9A mice: T322, S330, S332, S383, Y391, S396, T402, and T419 (Figure 7A). Two sites were more significantly phosphorylated in α1-containing GABA_*A*_Rs in S408/9A mice than in WT: T322 (WT: 0.54 ± 0.01, S408/9A: 0.63 ± 0.04, *p* = 0.0464, *n* = 5, unpaired *t*-test) and T419 (WT: 0.40 ± 0.03, S408/9A: 0.054 ± 0.05, *p* = 0.0380, *n* = 5, unpaired *t*-test) (Figure 7A). The same eight phosphorylation sites were detected in the α4 subtype in S408/9A mice as well (Figure 7B). Together, these results demonstrate that the mutation of S408/9 does not induce gross changes in the sites of basal phosphorylation within the β3 subunit in either the α1 or α4 receptor subtype. These results also suggest that the presence of α1α4-containing GABA_*A*_Rs in S408/9A mice is specifically due to the S408/9A mutation.

### The endoplasmic reticulum retention of the α4 subunit is decreased in S408/9A mice

Our results have demonstrated that the S408/9A mutation leads to altered subunit composition of α1-containing GABA_*A*_Rs in the forebrain (Figures 6B,C). The assembly of GABA_*A*_Rs from their component subunits occurs within the ER, a process that acts to limit the structural diversity of receptor subtypes that accumulate in the PM (Jacob et al., 2008). To understand the effects of the S408/9A mutation further, we investigated the expression of GABA_*A*_R subunits between subcellular compartments. We first subjected homogenized murine forebrain tissues to serial centrifugation from which cytoplasm, mitochondria, ER, and PM fractions were obtained (Suski et al., 2014; Smalley et al., 2020). These fractions were immunoblotted for marker proteins specific for each subcellular compartment. Enrichment of hsp90 in cytoplasmic fraction, hsp60 in mitochondria, calreticulin in ER, and n-cadherin in the PM (Supplementary Figure 3) validated our method of subcellular fractionation.

**FIGURE 8:**
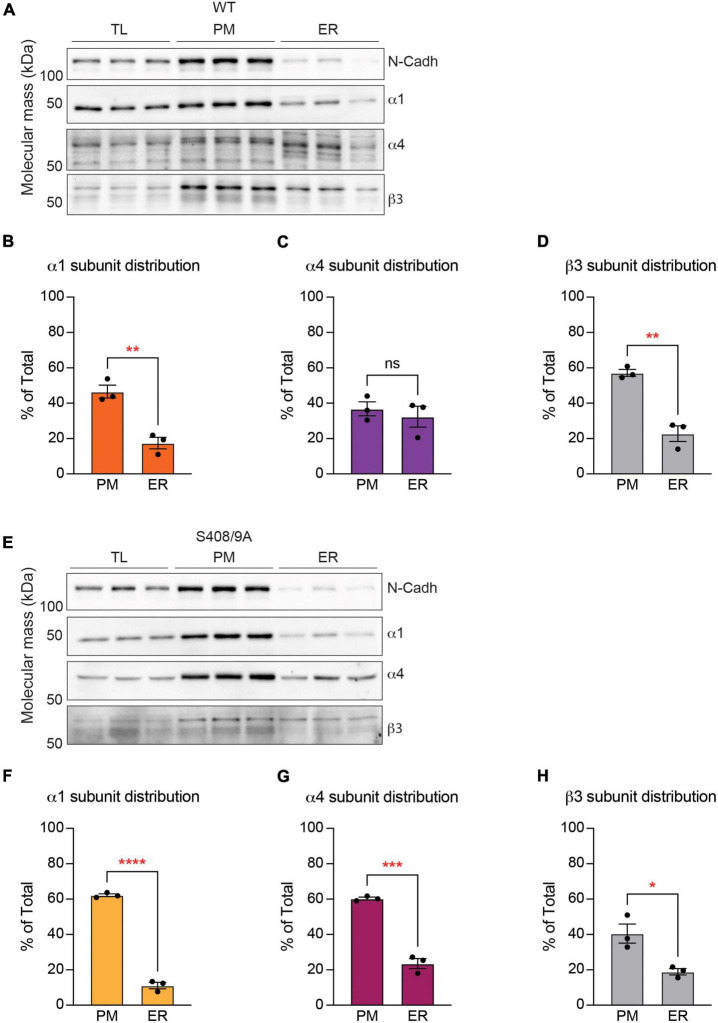
The S408/9A mutation selectively affects the distribution of the α4 subunit across subcellular compartments. **(A)** Immunoblots of total forebrain lysate (TL), PM, and ER fractions from WT animals were probed for N-cadh, α1, α4, and β3 subunits. **(B)** The distribution of each GABA_*A*_R subunit between PM and ER was calculated as a percentage of total expression in TL, PM, and ER. Higher percentage of the α1 subunit is expressed in the PM than in the ER (**p < 0.01, *n* = 3). **(C)** Similar levels of the α4 subunit are observed in the PM and ER (ns ≥ 0.05, *n* = 3). **(D)** The β3 subunit is more significantly enriched in the PM than in the ER (**p < 0.01, *n* = 3). **(E)** Levels of N-Cadh, α1, α4, and β3 subunits were assessed in immunoblots of TL, PM, and ER fractions from S408/9A animals. **(F–H)** In S408/9A animals, higher levels of the α1, α4, and β3 subunits are expressed in the PM than in the ER (*p < 0.05, ***p < 0.001, ****p < 0.0001, *n* = 3).

We then subjected purified total lysate (TL), PM, and ER fractions to SDS-PAGE and probed for n-cadherin and the α1, α4, and β3 subunits. The distribution of each GABA_*A*_R subunit between the PM and ER was calculated as a percentage of total expression in TL, PM, and ER. In WT mice, the α1 subunit immunoreactivity was significantly higher in the PM than in the ER (Figures 8A,B; PM: 46.57 ± 3.63%, ER: 17.43 ± 3.26%, *p* = 0.0040, *n* = 3, unpaired *t*-test). In contrast, similar amounts of the α4 subunit were found in the PM and the ER (Figures 8A,C; PM: 36.81 ± 3.94%, ER: 32.36 ± 5.90%, *p* = 0.5641, *n* = 3, unpaired *t*-test). Similarly to the α1 subunit, the majority of the β3 subunit immunoreactivity was found in the PM (Figures 8A,D; PM: 57.16 ± 1.94%, ER: 22.79 ± 4.40%, *p* = 0.0020, *n* = 3, unpaired *t*-test). In subcellular fractions prepared from S408/9A mice, all three subunits were more significantly expressed in the PM than in the ER: α1 subunit (PM: 62.18 ± 0.82%, ER: 11.11 ± 1.81%, *p* < 0.0001, *n* = 3, unpaired *t*-test), α4 subunit (PM: 60.30 ± 0.78%, ER: 23.56 ± 2.82%, *p* = 0.0002, *n* = 3, unpaired *t*-test), and β3 subunit (PM: 40.51 ± 5.39, ER: 18.93 ± 1.80, *p* = 0.0191, *n* = 3, unpaired *t*-test) (Figures 8E–H).

**FIGURE 9:**
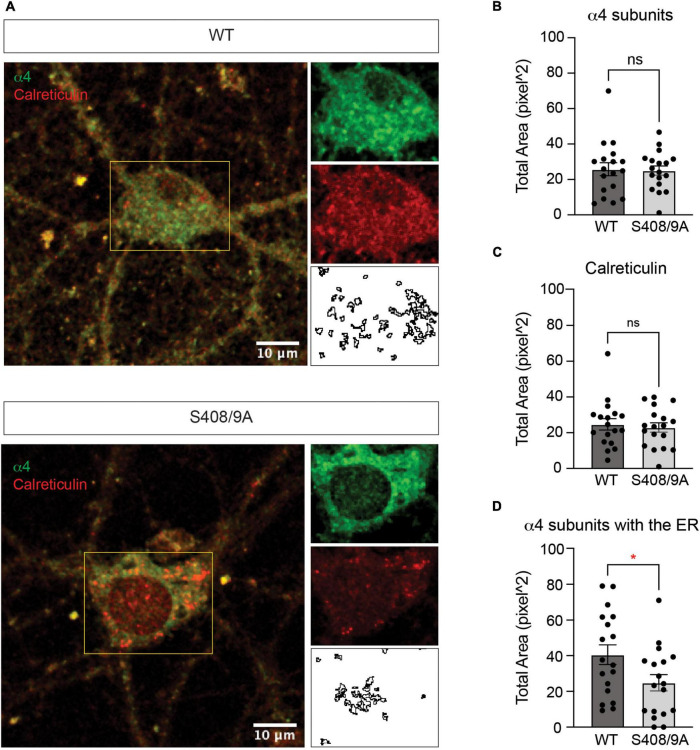
Colocalization of the α4 subunit with calreticulin is reduced in cultured neurons from S408/9A mice. **(A)** Representative images of the soma of DIV21 neurons cultured from WT and S408/9A mice stained with the α4 subunit and calreticulin antibodies are shown. Individual channels and areas of colocalization for each cell are shown on the right. **(B)** Total area of the α4 subunit staining in the soma is not affected by the S408/9A mutation (ns ≥ 0.05, *n* = 18 cells from three individually prepared cultures). **(C)** There is no significant difference in calreticulin staining in the soma between WT and S408/9A cultures (ns ≥ 0.05, *n* = 18 cells from three individually prepared cultures). **(D)** Colocalized area of the α4 subunit with calreticulin is reduced in S408/9A cultures when compared to WT cultures (*p ≤ 0.05, *n* = 18 cells from three individually prepared cultures).

To examine the effects of the S408/9A mutation on ER accumulation of GABA_*A*_R subunits further, we prepared primary cultured mixed cortical and hippocampal neurons from WT and S408/9A mice. We used immunocytochemistry to visualize the α4 subunit with calreticulin around the soma (Figure 9A). We found that there is no significant difference in the total area of the α4 subunit puncta (WT: 25.83 ± 3.67 pixel^2^, S408/9A: 25.02 ± 2.59 pixel^2^, *p* = 0.8577, *n* = 18, unpaired *t*-test) or calreticulin alone (WT: 24.74 ± 3.17 pixel^2^, S408/9A: 22.93 ± 2.63 pixel^2^, *p* = 0.6630, *n* = 18, unpaired *t*-test) between the two genotypes (Figures 9B,C). However, the total area of the α4 subunit and calreticulin co-localization is significantly reduced in S408/9A cells (Figure 9D; WT: 40.57 ± 5.56 pixel^2^, S408/9A: 24.84 ± 4.54 pixel^2^, *p* = 0.0352, *n* = 18, unpaired *t*-test). This is consistent with the results from the immunoblots of the PM and ER fractions (Figure 8) and suggests that there is less accumulation of the α4 subunit in the ER in S408/9A mice than in WT mice.

## Discussion

In the forebrain, phasic inhibition is mediated by GABA_*A*_Rs that contain the α1 subunit whereas tonic inhibition is dependent upon those assembled from the α4 subunit, and these populations of GABA_*A*_Rs are often co-expressed in neurons (Fritschy et al., 1998; Pirker et al., 2000). Here, we have begun to evaluate how the endogenous α1 and α4 subunits are segregated into distinct receptor subtypes. To do so, we have developed methods to isolate native α1- and α4-containing GABA_*A*_Rs from the PM and evaluated their subunit composition and binding proteins using BN-PAGE coupled with quantitative mass spectrometry.

Our approach revealed that affinity purified GABA_*A*_Rs containing the α1 subunit exhibited a native molecular mass of 250 kDa, which is consistent with the pentameric structure of GABA_*A*_Rs. Quantitative analysis of the 250 kDa species showed that α1-containing receptors contain the α1–3, β1–3, *γ*2–3, and δ subunits but not the α4 or α5 subunit. Based on the relative abundance, the subunit composition of endogenous α1-containing GABA_*A*_Rs was determined to be α1β3*γ*2, which has previously been found to be the most commonly encountered subunit composition in the brain (Olsen and Sieghart, 2009). Out of the α subunit isoforms, only the α2 and α3 subunits were significantly enriched with the α1 subunit when compared to control. This is consistent with previous studies that suggest the α1–3 subunits as synaptic α subunit variants (Brünig et al., 2002). Compared to the α1 subunit, lower levels of the α2 and α3 subunits were detected. Thus, the majority of synaptic GABA_*A*_Rs are likely to contain one or more copies of the α1 subunit. Such preferential assembly may result from the α1 subunit being expressed at 5–20-fold higher levels than other α subunit variants in the rodent forebrain (Chen et al., 2012). Notably, the δ subunit was detected as a significant binding partner of the α1 subunit in our purifications. While this result was unexpected, this is consistent with earlier studies suggesting that the δ subunit co-purifies with the α1, α3, β2–3, and *γ*2 subunits (Mertens et al., 1993). Moreover, receptors containing both the α1 and δ subunits have been suggested to be principal mediators of tonic inhibition in hippocampal interneurons (Fritschy and Mohler, 1995; Glykys et al., 2007).

The subunit composition of GABA_*A*_Rs assembled from the α4 subunit was explored using a similar approach involving affinity purification with the α4 subunit antibody and BN-PAGE. These receptors also exhibited a native molecular mass of 250 kDa. Quantitative analysis revealed the significant presence of only the α4 and β3 subunits in this 250 kDa species. No other α, β, or *γ* subunit variants or the δ subunit was significantly enriched. While a major limitation of our experimental approach is that we purify GABA_*A*_Rs *en masse* and thus low-abundance species may be overlooked, GABA_*A*_Rs composed of the α and β subunits only have been shown to mediate tonic inhibition in hippocampal pyramidal neurons (Mortensen and Smart, 2006). In fact, it is estimated that up to 10% of extrasynaptic GABA_*A*_Rs in hippocampal pyramidal neurons are αβ-containing receptors (Mortensen and Smart, 2006). Likewise, studies using immunoprecipitation coupled with immunoblotting suggest that more than 50% of α4-containing GABA_*A*_R in the brain are composed of the α and β subunits only (Bencsits et al., 1999).

The current dogma states that α4βδ is the most common subunit combination for GABA_*A*_Rs mediating tonic inhibition (Nusser and Mody, 2002; Wei et al., 2003; Peng et al., 2004; Jia et al., 2005). However, to our surprise, we did not detect a significant enrichment of the δ subunit in our purifications of α4-containing GABA_*A*_Rs. While neurons may assemble this receptor subtype in distinct brain regions such as the thalamus and DGGCs (Sur et al., 1999; Wei et al., 2003), our results suggest that α4β3 is the dominant subunit composition of α4-containing GABA_*A*_Rs in the adult mouse forebrain. This is consistent with a previous study that showed that only 7% of the α4-containing GABA_*A*_Rs contain the δ subunit (Bencsits et al., 1999). Given the sensitivity of δ-containing GABA_*A*_Rs to modulation by neurosteroids (Stell et al., 2003), the expression of α4δ-containing GABA_*A*_Rs may be induced by these endogenous modulators of GABAergic inhibition.

In addition to the 250 kDa species, a larger species of 720 kDa was seen in the purification of α1-containing GABA_*A*_Rs from the PM. Analysis of this larger species revealed that the α1 subunit co-purifies with 93 proteins, with spectrin isoforms (Sptan1, Sptbn1, and Sptbn2) making up more than 70% of the stable multiprotein complex. Spectrins are cytoskeletal integrators that bind to actin, microtubules, and intermediate filaments to provide structural support in cells (Liem, 2016). They are heterodimeric proteins that contain one α and one β isoforms – most spectrin molecules found in the brain are composed of αII-spectrin (encoded by Sptan1) and βII-spectrin (encoded by Sptbn1) (Machnicka et al., 2014; Liem, 2016). With each spectrin isoform at around 240 kDa, the high molecular weight species of α1-containing GABA_*A*_Rs at around 720 kDa is expected to be the pentameric GABA_*A*_Rs with dimeric spectrins. In neurons, spectrins form structures with ankyrins (Ank2/3), actin (Actn1), and adducin (Add1/3) (Xu et al., 2013; Han et al., 2017), all of which are significantly detected with the α1 subunit in low abundance. Together these proteins anchor cell surface transmembrane proteins to the cytoskeleton (Xu et al., 2013; Han et al., 2017).

Among the low-abundance proteins co-purifying with the α1 subtype is Kcc2 (encoded by Slc12a5), which is a neuronal membrane protein whose expression and function are critical for synaptic inhibitory transmission (Moore et al., 2017). The immunoblot of Kcc2 showed a band between 242 and 480 kDa and another between 480 and 720 kDa with a smear across 720 kDa. Since Kcc2 exists as monomers at around 140 kDa or as dimers at around 280 kDa (Smalley et al., 2020), the two bands would represent pentameric α1-containing GABA_*A*_Rs with monomeric and dimeric Kcc2, respectively. Lastly, gephyrin was detected with the α1 subunit at around 1,048 kDa. While a single gephyrin molecule is around 93 kDa, gephyrin forms hexagonal lattices to stabilize GABA_*A*_Rs and glycine receptors in the PM (Saiyed et al., 2007). Thus, it is reasonable that its combined molecular weight with pentameric GABA_*A*_Rs is higher than 720 kDa. As the major inhibitory scaffolding protein, gephyrin was found to associate with endogenous α2- and *γ*2-containing GABA_*A*_Rs as well (Nakamura et al., 2016; Ge et al., 2018). Other notable binding proteins of α1 subunit that were also identified to interact with the α2 and *γ*2 subunits by mass spectrometry include membrane proteins that regulate action potentials and membrane excitability in neurons: voltage-gated potassium channels (Kcna2 and Kcnb1), sodium/bicarbonate cotransporter (Slc4a10), and sodium channel (Scn2a1) (Nakamura et al., 2016; Ge et al., 2018).

Recently, two proteins - Lhfpl4 and Shisa7 - have been reported to bind to GABA_*A*_Rs and regulate their membrane trafficking (Davenport et al., 2017; Han et al., 2019; Yamasaki et al., 2017). However, we did not detect significant levels of either protein co-purifying with the α1 subunit. Our failure to detect these proteins may reflect that we utilized purified plasma membranes as a starting material while those on Lhfpl4 and Shisa7 used brain extracts or synaptosomal fractions. Alternatively, differences between the extraction buffers and/or detergents employed may also be of significance.

Interestingly, we found that purified α4-containing GABA_*A*_Rs also form a stable multiprotein complex at around 720 kDa and that it contains 19 proteins. Among the proteins that were significantly enriched with the α4 subunit, the most abundant is titin, which is known primarily as a protein that provides elasticity in muscles (Freundt and Linke, 2019). Its expression and function in neurons have not been determined, however. Another binding protein of the α4 subunit is desmoplakin, which interacts with n-cadherin, a cell adhesion protein in the membrane (Tanaka et al., 2012). Interestingly, desmoplakin was found to associate with the *γ*2 subunit as well (Ge et al., 2018). For the α4 subunit, the relative abundance of each protein is comparable, suggesting that the 720 kDa species would be a summation of pentameric α4-containing GABA_*A*_Rs with these low-abundance proteins. We also noted that neither gephyrin nor spectrin isoforms co-purified with the α4 subunit. However, two proteins were detected with both the α1 and α4 subunits in our findings: Myo5a and Vdac2. Myo5a has been implicated in subcellular localization of gephyrin (Fuhrmann et al., 2002) and Vdac2 has been found to associate with GABA_*A*_Rs (Bureau et al., 1992; Darbandi-Tonkabon et al., 2004).

Collectively, our experiments suggest that at steady state, neurons segregate the α1 and α4 subunits into biochemically distinct receptor subtypes. Moreover, analysis of the binding proteins of the two receptor subtypes suggests that GABA_*A*_Rs containing the α1 subunit are associated with core components of the postsynaptic inhibitory scaffold, which is consistent with their role in mediating phasic inhibition. In contrast, those assembled from the α4 subunit are not associated with inhibitory scaffolding proteins. The significant difference in the components of stable protein complexes between the two GABA_*A*_R subtypes further strengthens the suggestion that the α1 and α4 subunits are sorted into separate populations.

Interestingly, the β3 subunit is significantly enriched in both α1 and α4 subtypes. Furthermore, the β3 subunit associated with the α1 subunit exhibits higher phosphorylation of S408/9 than that in the α4 subtype. Previous studies have shown that S408/9 phosphorylation acts to modify membrane trafficking of GABA_*A*_Rs and promotes their residence time in the PM (Kittler et al., 2005; Vien et al., 2015). Moreover, aberrant levels of S408/9 phosphorylation have been observed in mouse models of status epilepticus and fragile X syndrome (FXS) (Terunuma et al., 2008; Vien et al., 2015). We found that disrupting the phospho-regulation of S408/9 compromises the segregation of the α1 and α4 subunits into distinct receptor subtypes, leading to the production of α1α4-containing GABA_*A*_Rs. In addition to the α4 subunit, the amount of the α5 subunit detected with the α1 subunit was significantly enhanced in the mutants. The presence of the α4 and α5 subunits altered the proportion of receptors occupied by the α1 subunit, suggesting that α1-containing GABA_*A*_Rs in S408/9A mice may only have one copy of the α1 subunit. Based on the relative abundance of subunits detected, α1β2/3*γ*2 is still likely the subunit composition of α1-containing GABA_*A*_Rs in the mutant mice. Functional studies have shown that the spontaneous amplitude of inhibitory synaptic currents is increased in S408/9A mice, while tonic currents are decreased (Vien et al., 2015). Thus, the presence of α1α4-containing receptors in S408/9A mice may contribute to these reciprocal modifications in phasic and tonic inhibition.

For the binding proteins, the mutation did not interfere with the interactions between α1-containing GABA_*A*_Rs and spectrin isoforms (Sptan1, Sptbn1, and Sptbn2), Kcc2, or gephyrin. This suggests that the stabilization and synaptic targeting of the α1 subunit are still intact even with the presence of the α4 and α5 subunits. It also suggests that the novel α1α4-containing GABA_*A*_Rs may be localized to the synaptic areas. In an animal model of FXS, in which the level of S408/9 phosphorylation is significantly altered compared to healthy mice, the α4 subunit selective agonist Ro15-4513 potentiates synaptic currents (Vien et al., 2015; Modgil et al., 2019). In addition, the α4 subunit is found in synaptic areas (Zhang et al., 2017), although the molecular mechanism for the mis-trafficking has not been revealed. It is possible that aberrant S408/9 phosphorylation leads to the formation of α1α4-containing GABA_*A*_Rs and as a result, the α4 subunit is mis-trafficked to synapses.

While the S408/9A mutation did not modify the total level of the α1, α4, or β3 subunit and did not induce any gross changes in the phosphorylation of the β3 subunit, it did reduce the levels of the α4 subunit in the ER and increase its accumulation in the PM. The steady state levels of GABA_*A*_R subunits within the ER are dependent upon oligomerization into transport-competent hetero-oligomers, as well as their rates of retrograde transport and subsequent proteasomal degradation (Jacob et al., 2008; Luscher et al., 2011). Thus, S408/9A are likely to induce the assembly of the α4 subunit into hetero-oligomers, promoting their subsequent transport from the ER to PM and contributing to changes in GABAergic inhibition (Vien et al., 2015). Our study also identified several novel phosphorylation sites on the intracellular loop of the β3 subunit - T322, S330, S332, Y391, S396, T402, and T419. Phosphorylated S332 was only detected on the β3 subunit that is associated with the α1 subunit in WT animals. However, it was detected in both α1 and α4 subtypes in S408/9A animals. Additionally, T322 and T419 were more significantly phosphorylated in the α1 subtype in the mutants than in WT. Further investigation into these novel phosphorylation sites may provide additional insights into mechanisms of GABA_*A*_R regulation.

In summary, our results have provided insights into the mechanisms neurons utilize to orchestrate the assembly of GABA_*A*_R subunits into distinct receptor subtypes, which is required to support phasic and tonic inhibition. They also suggest that receptor assembly is subject to dynamic modulation via S408/9 in the β3 subunit, which are key sites for phosphorylation-dependent regulation of surface GABA_*A*_R expression. How S408/9 phosphorylation regulates the segregation of the α1 and α4 subunits into distinct receptor subtypes is an open question, but our results suggest that this process may have an impact on the β3 subunit’s half-life in the ER, a key determinant for subunit oligomerization, ER exit, and transport to the PM (Nakamura et al., 2016). Thus, cell signaling molecules that regulate S408/9 phosphorylation, such as neurosteroids (Adams et al., 2015; Modgil et al., 2017, 2019; Parakala et al., 2019), may exert long lasting effects on neuronal activity by regulating GABA_*A*_R assembly.

## Materials and methods

### Animals

WT C57BL/6 and S408/9A animals were kept in a temperature-controlled room on a 12-h light/dark cycle and fed *ad libitum* with 2 cage changes per week. S408/9A animals were generated by gene targeting in murine ES cells as previously described (Vien et al., 2015, 2022). 8–12 weeks old male and female S408/9A homozygous mice and WT littermate controls were used for all biochemistry experiments. For immunocytochemistry experiments, P0 WT and S408/9A homozygous pups were used. All experimental procedures were approved by the Tufts University Institutional Animal Care and Use Committee (IACUC).

### Antibodies

The following antibodies were used for immunoprecipitation (IP), western blot (WB), or immunocytochemistry (ICC): Gabra1 (mouse, NeuroMab clone N95/35, IP), Gabra1 (rabbit, Abcam ab33299, WB 1:1000, ICC 1:1000), Gabra4 (mouse, NeuroMab clone N398A/34, IP, ICC 1:500), Gabra4 (rabbit, PhosphoSolutions 845A-GA4C, WB 1:1000), Gabrb3 (rabbit, PhosphoSolutions 863A-GB3C, WB 1:1000), p-S408/9 (PhosphoSolutions p1130-4089, WB 1:1000), Sptan1 (rabbit, Cell Signaling 2122S, WB 1:1000), Sptbn1 (rabbit, Abcam ab72239, WB 1:1000), Sptbn2 (rabbit, Proteintech 55107-1-AP, WB 1:1000), Erc2 (rabbit, Abcam ab170862, WB 1:1000), Kcc2 (rabbit, Millipore 07-432, WB 1:2000), Gephyrin (rabbit, Cell Signaling 14304S, WB 1:1000), N-Cadherin (mouse, Cell Signaling 14215S, WB 1:1000), Calreticulin (rabbit, Cell Signaling 12238S, WB 1:1000, ICC 1:500), Hsp60 (rabbit, Cell Signaling 12165S, WB 1:1000), Hsp90 (rabbit, Cell Signaling 4877S, WB 1:1000), GAPDH (mouse, Santa Cruz sc-32233, WB 1:5000), Goat anti-mouse Alexa Fluor 488 (Thermo Fisher A11029, ICC 1:1000), Goat anti-rabbit Alexa Fluor 568 (Thermo Fisher A11011, ICC 1:1000), Donkey anti-mouse conjugated HRP (Jackson ImmunoResearch 715-035-150, WB 1:5000-7000), Donkey anti-rabbit conjugated HRP (Jackson ImmunoResearch 711-035-152, WB 1:2500-7000).

### Plasma membrane isolation

Fresh forebrain tissues from 8–12 weeks old male and female mice were dissected in ice-cold 1× phosphate-buffered saline (PBS) and collected in starting buffer (225 mM mannitol, 75 mM sucrose, and 30 mM Tris-HCl, pH 7.4) as described previously (Smalley et al., 2020). Forebrain tissues from 7 animals were pooled and homogenized in isolation buffer [225 mM mannitol, 75 mM sucrose, 0.5% BSA (w/v), 0.5 mM EGTA, and 30 mM Tris-HCl, pH 7.4] supplemented with a protease inhibitor (cOmplete mini, EDTA-free Protease Inhibitor Cocktail, Roche 11836170001) and a phosphatase inhibitor (PhosSTOP, Roche 4906837001) on ice using 14 strokes of a Dounce homogenizer. The homogenates were subjected to serial centrifugation to isolate purified PM and ER fractions. The PM and ER fractions were solubilized in Triton lysis buffer [150 mM NaCl, 10 mM Tris, 0.5% Triton X-100 (v/v), pH 7.5] supplemented with protease and phosphatase inhibitors.

### Immunoprecipitation

Protein G Dynabeads (Thermo Fisher 10004D) were washed three times with 1× PBS with 0.05% Tween-20 (v/v; 0.05% PBS-T). The beads were resuspended in 0.05% PBS-T and incubated overnight at 4°C with antibodies for the target protein or non-immune mouse IgG (Jackson ImmunoResearch 015-000-003). The beads were washed twice with 0.2 M triethanolamine (TEA; pH 8.2) and incubated for 30 min with 40 mM dimethyl pimelimidate in TEA at room temperature for antibody cross-linking. The beads were incubated for 15 min with 50 mM Tris (pH 7.5) at room temperature and washed three times with 0.05% PBS-T before resuspension in solubilized PM fractions and incubation overnight at 4°C. The beads were washed three times with 0.05% PBS-T and eluted with soft elution buffer [0.2% SDS (w/v), 0.1% Tween-20 (v/v), 50 mM Tris-HCl, pH 8.0] for BN-PAGE as outlined previously (Antrobus and Borner, 2011; Smalley et al., 2020).

### BN-PAGE

Samples were diluted in 4× NativePAGE sample buffer and 5% G-250 sample additive (Invitrogen BN2008) and loaded onto 4–16% NativePAGE gradient gels (Invitrogen BN1002) as detailed previously (Smalley et al., 2020). Gels were run for approximately 2 h in anode and cathode buffers (NativePAGE running buffer, Invitrogen BN2001; NativePAGE cathode buffer additive, Invitrogen BN2002). For immunoblotting, proteins were transferred to PVDF membranes overnight at 4°C. The membranes were fixed in 8% acetic acid (v/v), washed with water, and air-dried before de-staining with 100% methanol. The membranes were then blocked in 5% milk (w/v) in tris-buffered saline with 0.1% Tween-20 (v/v; TBS-T) for 1 h, washed with TBS-T, and incubated with primary antibodies diluted in TBS-T overnight at 4°C. After washing with TBS-T, the membranes were incubated with secondary antibodies diluted in TBS-T for 1 h at room temperature. Protein bands were visualized with SuperSignal West Dura Extended Duration Substrate (Thermo Scientific 34075) and imaged using a ChemiDoc MP (Bio-Rad). Images were cropped at the bottom (below 150 kDa) to remove the dye front, which contains unbound colloidal Coomassie blue. For liquid chromatography coupled with tandem mass spectrometry (LC-MS/MS), the gels were fixed in fixing solution [50% ethanol (v/v), 10% acetic acid (v/v)], washed in ethanol solution [30% ethanol (v/v)], washed in water, then stained overnight with colloidal Coomassie blue (CCB; Sigma-Aldrich G1041). The gels were de-stained in water, imaged, and regions around the bands of interest were excised for LC-MS/MS.

### SDS-PAGE

Protein concentration was measured using a Bradford assay (Bio-Rad 5000006). Samples were diluted in 2× sample buffer (Sigma-Aldrich S3401) and 20–50 μg of protein was loaded onto 10% polyacrylamide gels. After separation by SDS-PAGE, proteins were transferred to nitrocellulose membranes overnight at 4°C. The membranes were blocked in 5% milk or BSA, incubated with primary and secondary antibodies, and imaged as described above (Smalley et al., 2020; Kontou et al., 2021).

### Primary neuronal culture

P0 pups were anesthetized on ice and immediately euthanized by decapitation. The brains were quickly removed, and cortical and hippocampal tissues were dissected in ice-cold Hank’s buffered salt solution (HBSS; Thermo Fisher 14185052) with 10 mM HEPES. The tissues were trypsinized and titurated in Neurobasal-A media (Thermo Fisher 10888022) containing 2% B27 (v/v; Thermo Fisher 17504044), 1% GlutaMAX (v/v; Thermo Fisher 35050061), and 1% Penicillin/Streptomycin (v/v; Thermo Fisher 15140122). Dissociated neurons were filtered through a 40 μm nylon mesh strainer (Thermo Fisher 22363547) and counted using a hemocytometer. Cells were plated on poly-L-lysine (PLL)-coated 13 mm coverslips in 24-well plates at a density of 100–125 K cells/well in media. At days *in vitro* (DIV) 21, cells were fixed in 4% paraformaldehyde in PBS (v/v) for 10 min at room temperature and placed in PBS at 4°C (Kontou et al., 2021).

### Immunocytochemistry

Fixed cultures were permeabilized for 1 h in blocking solution [3% BSA (w/v), 10% normal goat serum (v/v), 0.2 M glycine in PBS, 0.1% Triton-X100 (v/v)] (Kontou et al., 2021). Coverslips were incubated with primary antibodies diluted in blocking solution for 1 h at room temperature. After a brief wash with PBS, the coverslips were incubated with fluorophore-conjugated secondary antibodies diluted in blocking solution for 1 h at room temperature. The coverslips were then washed in PBS, briefly dipped in DAPI ready-made solution (Sigma-Aldrich MBD0015), and mounted onto microscope slides with Fluoromount-G (SouthernBiotech 0100-01). The coverslips were imaged using a Nikon A1 confocal microscope (Nikon Instruments, Melville, NY, USA) using a 60× oil immersion objective lens. Laser settings were manually assigned for each fluorescent channel and images were acquired at the scan size of 1,024 × 1,024. Settings were kept the same between imaging sessions and between genotypes.

### Image analysis

For immunoblots, individual band intensity was quantified using densitometry on ImageJ (Version 1.53) normalized to GAPDH and further normalized to the corresponding control condition where appropriate. For immunocytochemistry, 18 neurons from 3 separate cultures per genotype were analyzed on FIJI (Version 2.3.0). The background was subtracted using the rolling ball radius of 50, and the images were smoothed with the median filter of 1.0. The total areas of individual protein staining were quantified using Analyze Particle with the range of 10–400 pixel units. For colocalization analysis, Image Calculator was used to find areas of overlap and Analyze Particle was used to obtain the total areas of colocalization (Kontou et al., 2021).

### LC-MS/MS

Peptides were extracted from gel pieces and the extracts were dried in a speed-vac for 1 h and stored at 4°C until analysis (Smalley et al., 2020). For analysis, each sample was loaded onto a nanoscale reversed-phase liquid chromatography capillary column packed with C18 silica beads. A gradient was formed between solvent A (97.5% water, 2.5% acetonitrile, and 0.1% formic acid) and increasing concentrations of solvent B (97.5% acetonitrile, 2.5% water, and 0.1% formic acid). Eluted peptides were subjected to nanospray ionization and then entered into an LTQ Orbitrap Velos Pro ion-trap mass spectrometer (Thermo Finnigan, San Jose, CA, USA). MS1 parameters were: resolution of 70 K, scan range of mass-to-charge ratio (m/z) of 85–2,000, charge-state screening parameters of +2 to +5, precursor ion isolation window of 2 m/z, and centroid acquisition mode. Eluting peptides were detected and the most intense were isolated using the Top 10 scan mode and fragmented by higher energy C-trap dissociation at the normalized collision energy of 35%. MS2 ions were analyzed by an Orbitrap mass spectrometer with the resolution of 17.5 K and the dynamic exclusion settings (repeat count: 1, repeat duration: 30 s, exclusion duration: 60 s, exclusion mass width: 10 ppm) to produce a tandem mass spectrum of specific fragment ions for each peptide.

### LC-MS/MS analysis

Peptide searches were performed as detailed previously (Smalley et al., 2020). Peptide sequences were determined by matching protein or translated nucleotide databases with the acquired fragmentation pattern using the MSGF+ (Kim and Pevzner, 2014). Raw mzXML files were used to search the UniProt mouse reference proteome (last modified May 4th 2020, containing 21,989 sequences), which contains the Thermo list of common contaminants. For phosphopeptide detection, a variable modification of mass units to serine, threonine, and tyrosine was included in the database searches. The resulting mzID files from the spectral searches were combined with mzXML files using MSnbase package in R (accessed July 20th 2020) and used to calculate peptide counts and the quantitative measurement: spectral index normalized to global intensity (SI_GI_). The SI_GI_ values for each protein detected by Gabra1 or Gabra4 immunoprecipitation were compared to non-immune IgG purifications by Welch’s *t*-test to identify significantly enriched proteins. For network analysis, the protein lists were compared against the latest version of the STRINGdb database (Szklarczyk et al., 2019). The interaction for each protein with Gabra1 or Gabra4 was imputed and network diagrams were constructed in R using the igraph package (accessed February 1st, 2019) and the nodes were scaled to the average SI_GI_ values for each protein. The highest scoring Gene Ontology Biological Process term was extracted for each protein using the mygene package (accessed July 29th, 2020). For PCA, The SI_GI_ values for proteins contained within each gel band were normalized by z-transformation using the ggfortify package in R. Only the significantly enriched proteins detected in all replicates were included in the analysis.

### Statistics

For LC-MS/MS data, significant proteins were detected by performing the Welch’s *t*-test between the samples and non-immune IgG purifications. Significance on immunoblots and ICC data was determined by performing the unpaired Student’s *t*-test on GraphPad Prism (Version 9.3.1). Varying levels of significance were denoted as the following: ns (*p* ≥ 0.05), * (*p* < 0.05), ^**^ (*p* < 0.01), ^***^ (*p* < 0.001), ^****^ (*p* < 0.0001).

## Data availability statement

The datasets presented in this study can be found in online repositories. The names of the repository/repositories and accession number(s) can be found below: http://www.proteomexchange.org/, PXD036295.

## Ethics statement

The animal study was reviewed and approved by Tufts University Institutional Animal Care and Use Committee (IACUC).

## Author contributions

CC performed the experiments, analyzed the data, and wrote the manuscript. JS, AL, and QR performed the experiments. CB and JD maintained the mouse colony and performed genotyping. SM and JS conceptualized the project and designed the experiments. CC, JS, PD, and SM edited the manuscript. All authors contributed to the article and approved the submitted version.
