# Supplementary material for: Analyzing the mechanisms that facilitate the subtype-specific assembly of γ-aminobutyric acid type A receptors
Source: Front Mol Neurosci. 2022 Oct 3;15:1017404. doi: 10.3389/fnmol.2022.1017404 (PMC9574402; doi:10.3389/fnmol.2022.1017404)
Supplement: Supplementary file 1 [file Data_Sheet_1.DOCX]

Supplementary Material

**Supplementary Figure 1. (A)** Purified PM, PM exposed to non-immune IgG or the α1 antibody (IP), and flow through samples (FT) were resolved on SDS-PAGE and probed for the α1 subunit. Immunoreactivity of the α1 subunit was detected at expected molecular weights of around 50 kDa in PM and α1 IP lanes but not in IgG lane. The level of the α1 subunit expression is reduced in FT sample of α1 IP compared to FT sample of IgG. This validates our approach for effective purification of endogenous α1-containing GABA_A_Rs. **(B)** Levels of the α4 subunit were assessed in PM, PM exposed to IgG or the α4 antibody (IP), and FT. Positive immunoreactivity of the α4 subunit was observed for PM and α4 IP lanes and the level of the α4 subunit expression is reduced in FT sample of α4 IP compared to IgG, suggesting effective purification of endogenous α4-containing GABA_A_Rs.

**Supplementary Figure 2. (A)** In the 720 kDa band of α1-containing GABA_A_Rs from S408/9A mice, 38 significantly enriched proteins were detected. Heatmap shows Sptan1, Sptbn1, and Sptbn2 are the most abundant proteins, suggesting that the mutation does not interfere with their interactions with the receptor subtype. **(B)** Breakdown of the binding proteins of the α1 subunit shows that more than 70% is spectrin isoforms. Proteins that constitute less than 2% of the total amount of proteins (28 proteins) are grouped together as ‘Other.’ **(C)** There is no significant difference in the amount of the α1 subunit detected in the 720 kDa bands between WT and S408/9A (ns ≥ 0.05, n = 5 replicates). **(D)** The α4-containing GABA_A_Rs in S408/9A mice only have 9 significantly enriched binding proteins, which are listed in the heatmap. Tchh is one protein that is significantly enriched in both α1 and α4 subtypes in S408/9A mice. **(E)** 33% of total protein detected with the α4 subtype is Loxl4, followed by 22% of Adamts15, 19% of Nod2, and 6% or less of each of the remaining 6 proteins. **(F)** There is no significant difference in the amount of the α4 subunit detected in the 720 kDa bands between the two genotypes, suggesting that the mutation does not affect the efficiency of our purification method (ns ≥ 0.05, n = 5 replicates).

**Supplementary Figure 3.** Subcellular fractions collected from forebrain tissues of WT mice were probed for marker proteins. Specific enrichment of hsp90 in cytoplasm (Cyto), hsp60 in mitochondria (Mito), calreticulin (Calr) in ER, and n-cadherin (N-Cadh) in PM show efficient fractionation of subcellular compartments.

**Supplementary Table 1.** Significantly enriched subunits detected in the 250 kDa bands. Proteins are listed in order of abundance (Average SI_GI_). Welch’s *t-*test was performed to calculate the p-values relative to non-immune IgG control. **(A)** α1-containing GABA_A_Rs in WT. **(B)** α4-containing GABA_A_Rs in WT. **(C)** α1-containing GABA_A_Rs in S408/9A. **(D)** α4-containing GABA_A_Rs in S408/9A.

**Supplementary Table 2.** Significantly enriched subunits detected in the 720 kDa bands. Proteins are listed in order of abundance (Average SI_GI_). Welch’s *t-*test was performed to calculate the p-values relative to non-immune IgG control. **(A)** α1-containing GABA_A_Rs in WT. **(B)** α4-containing GABA_A_Rs in WT. **(C)** α1-containing GABA_A_Rs in S408/9A. **(D)** α4-containing GABA_A_Rs in S408/9A.
