# Supplementary material for: Analyzing the mechanisms that facilitate the subtype-specific assembly of γ-aminobutyric acid type A receptors
Source: Front Mol Neurosci. 2022 Oct 3;15:1017404. doi: 10.3389/fnmol.2022.1017404 (PMC9574402; doi:10.3389/fnmol.2022.1017404)
Supplement: Supplementary file 2 [file Data_Sheet_2.PDF]

Table S1. Significantly enriched proteins detected in the 250 kDa bands of α1- and α4-containing GABA$_A$Rs in WT (**A-B**) and S408/9A (**C-D**) mice in order of abundance (Average SI$_{GI}$). Welch's *t*-test was performed to calculate the *p*-values relative to non-immune IgG control.

A. α1-containing GABA$_A$Rs in WT

Protein	Uniprot ID	Description	Average SI$_{GI}$	p-value
Gabra1	P62812	Gamma-aminobutyric acid receptor subunit alpha-1	0.008708068	3.17861E-05
Gabrb3	P63080	Gamma-aminobutyric acid receptor subunit beta-3	0.0035461	0.00257564
Gabrg2	P22723	Gamma-aminobutyric acid receptor subunit gamma-2	0.002431222	3.75554E-05
Gabrb2	P63137	Gamma-aminobutyric acid receptor subunit beta-2	0.001369792	0.00266997
Gabra3	P26049	Gamma-aminobutyric acid receptor subunit alpha-3	0.000734308	0.000931304
Gabrb1	P50571	Gamma-aminobutyric acid receptor subunit beta-1	0.00061952	0.00685247
Gabrd	P22933	Gamma-aminobutyric acid receptor subunit delta	0.000402412	0.002173593
Gabra2	P26048	Gamma-aminobutyric acid receptor subunit alpha-2	0.000345286	0.009420038
Gabrg3	P27681	Gamma-aminobutyric acid receptor subunit gamma-3	0.000103759	0.004974281

B. α4-containing GABA$_A$Rs in WT

Protein	Uniprot ID	Description	Average SI$_{GI}$	p-value
Gabra4	Q9D6F4	Gamma-aminobutyric acid receptor subunit alpha-4	0.004562909	0.00488842
Gabrb3	P63080	Gamma-aminobutyric acid receptor subunit beta-3	0.001010096	0.001305036

C. α1-containing GABA$_A$Rs in S408/9A

Protein	Uniprot ID	Description	Average SI$_{GI}$	p-value
Gabra1	P62812	Gamma-aminobutyric acid receptor subunit alpha-1	0.006703812	0.015536925
Gabrg2	P22723	Gamma-aminobutyric acid receptor subunit gamma-2	0.003438939	0.000642812
Gabrb2	P63137	Gamma-aminobutyric acid receptor subunit beta-2	0.002891284	0.00012474
Gabrb3	P63080	Gamma-aminobutyric acid receptor subunit beta-3	0.002621153	2.73161E-05
Gabra3	P26049	Gamma-aminobutyric acid receptor subunit alpha-3	0.000984037	0.003788105
Gabrb1	P50571	Gamma-aminobutyric acid receptor subunit beta-1	0.000705577	0.008121264
Gabra4	Q9D6F4	Gamma-aminobutyric acid receptor subunit alpha-4	0.000660294	0.035204646
Gabra5	Q8BHJ7	Gamma-aminobutyric acid receptor subunit alpha-5	0.000617457	0.043039436
Gabrd	P22933	Gamma-aminobutyric acid receptor subunit delta	0.000224148	0.036723032

D. α4-containing $GABA_ARs$ in S408/9A

Protein	Uniprot ID	Description	Average SI_{GI}	p-value
Gabra4	Q9D6F4	Gamma-aminobutyric acid receptor subunit alpha-4	0.006300639	0.001087995
Gabrb3	P63080	Gamma-aminobutyric acid receptor subunit beta-3	0.001854076	0.004516503
