# Supplementary material for: Analyzing the mechanisms that facilitate the subtype-specific assembly of γ-aminobutyric acid type A receptors
Source: Front Mol Neurosci. 2022 Oct 3;15:1017404. doi: 10.3389/fnmol.2022.1017404 (PMC9574402; doi:10.3389/fnmol.2022.1017404)
Supplement: Supplementary file 3 [file Data_Sheet_3.PDF]

Table S2. Significantly enriched proteins detected in the 720 kDa bands of α1- and α4-containing $GABA_A$Rs in WT (**A-B**) and S408/9A (**C-D**) mice in order of abundance (Average SI_{GI}). Welch's *t*-test was performed to calculate the *p*-values relative to non-immune IgG control.

A. α1-containing $GABA_A$Rs in WT

Protein	Uniprot ID	Description	Average SI_{GI}	p-value
Sptan1	P16546	Spectrin alpha chain, non-erythrocytic 1	0.247584903	0.000808768
Sptbn1	Q62261	Spectrin beta chain, non-erythrocytic 1	0.107805882	0.000460287
Sptbn2	Q68FG2	Spectrin beta chain, non-erythrocytic 2	0.02921047	4.0898E-05
Myh10	Q61879	Myosin-10	0.025915281	2.51965E-05
Myo5a	Q99104	Unconventional myosin-Va	0.01220778	0.004384183
Sptb	P15508	Spectrin beta chain, erythrocytic	0.009290474	0.00047213
Ank2	Q8C8R3	Ankyrin-2	0.008712587	1.41908E-05
Synpo	Q8CC35	Synaptopodin	0.00605616	0.000103949
Actn1	Q7TPR4	Alpha-actinin-1	0.006008638	0.005672924
Erc2	Q6PH08	ERC protein 2	0.005749661	0.002154232
Myo18a	Q9JMH9	Unconventional myosin-XVIIIa	0.004778657	0.000122877
Srcin1	Q9QWI6	Src kinase signaling inhibitor 1	0.004577605	6.93503E-05
Ina	P46660	Alpha-internexin	0.004349712	4.24269E-06
Map6	Q7TSJ2	Microtubule-associated protein 6	0.003336494	0.011335518
Nefm	P08553	Neurofilament medium polypeptide	0.003223048	0.038754097
Ppp1r9b	Q6R891	Protein phosphatase 1, regulatory subunit 9b	0.002264664	7.69211E-06
Slc4a10	Q5DTL9	Sodium-driven chloride bicarbonate exchanger	0.002212256	0.030670492
Slc12a5	Q91V14	Solute carrier family 12 member 5	0.001851388	0.042984818
Dbn1	Q9QXS6	Drebrin 1	0.001568317	0.001689845
Gnao1	P18872	Guanine nucleotide-binding protein G(o) subunit alpha	0.001557775	0.003385973
Scn2a1	B1AWN6	Sodium channel protein type 2 subunit alpha	0.001516454	7.00766E-06
Myo6	Q64331	Unconventional myosin-VI	0.001509624	0.003013816
Homer1	Q9Z2Y3	Homer protein homolog 1	0.001380287	1.82729E-06
Map4	P27546	Microtubule-associated protein 4	0.001379918	0.009993627
Add1	Q9QYC0	Alpha-adducin	0.001367912	0.019175378

Vdac2	Q60930	Voltage-dependent anion-selective channel protein 2	0.001338048	3.71423E-06
Dmtn	Q9WV69	Dematin	0.001260508	0.000128397
Cltc	Q68FD5	Clathrin heavy chain 1	0.001243217	0.011261358
Grm3	Q9QYS2	Metabotropic glutamate receptor 3	0.001205569	0.030540357
Syt7	Q9R0N7	Synaptotagmin-7	0.001202304	0.020997101
Shank3	Q4ACU6	SH3 and multiple ankyrin repeat domains protein 3	0.001126041	0.039512837
Akap5	D3YVF0	A-kinase anchor protein 5	0.001117415	0.000237926
Tjp1	P39447	Tight junction protein ZO-1	0.000963132	0.002290122
Ppp1r9a	H3BJD6	Protein phosphatase 1, regulatory subunit 9A	0.000874281	0.004023758
Myo1b	P46735	Unconventional myosin-Ib	0.00086431	0.000304233
Dock9	Q8BIK4	Dedicator of cytokinesis protein 9	0.000849499	0.040507605
Fhl3	Q9R059	Four and a half LIM domains 3	0.000838105	0.002645776
Vapa	Q9WV55	Vesicle-associated membrane protein-associated protein A	0.000830499	0.024631066
Gabra1	P62812	Gamma-aminobutyric acid receptor subunit alpha-1	0.000795845	0.002481867
Cyfip2	Q5SQX6	Cytoplasmic FMR1-interacting protein 2	0.000791453	0.007415847
Gabrg2	P22723	Gamma-aminobutyric acid receptor subunit gamma-2	0.000782435	0.040956793
Ank3	G5E8K5	Ankyrin-3	0.000763627	0.010368917
Fsip2	A2ARZ3	Fibrous sheath-interacting protein 2	0.000694718	8.63547E-06
Atp2a2	O55143	Sarcoplasmic/endoplasmic reticulum calcium ATPase 2	0.000664547	0.030355358
Dock10	Q8BZN6	Dedicator of cytokinesis protein 10	0.000651508	0.024217402
Sdc4	O35988	Syndecan-4	0.000649871	0.000714868
Nrxn1	Q9CS84	Neurexin-1-beta	0.000639485	0.003973722
Atp5a1	Q03265	ATP synthase subunit alpha, mitochondrial	0.000566116	0.028294324
Camk2a	P11798	Calcium/calmodulin-dependent protein kinase type II subunit alpha	0.000565839	0.00522703
Camkv	Q3UHL1	CaM kinase-like vesicle-associated protein	0.000565485	0.038464337
Gphn	Q8BUV3	Gephyrin	0.000546955	0.001679289
Lman1	Q9D0F3	Protein ERGIC-53	0.000536957	0.030099358
Gabbr2	Q80T41	Gamma-aminobutyric acid type B receptor subunit 2	0.000507416	0.021954193
Wasf1	Q8R5H6	Wiskott-Aldrich syndrome protein family member 1	0.000497928	0.013387617
Ablim2	Q8BL65	Actin-binding LIM protein 2	0.000451152	0.010383215
Trmt13	Q8BYH3	tRNA:m(4)X modification enzyme TRM13 homolog	0.000450967	0.016000525

Fmnl2	A2APV2	Formin-like protein 2	0.000444191	0.015021473
Etl4	A2AQ25	Sickle tail protein	0.000418614	0.010582029
Add3	Q9QYB5	Gamma-adducin	0.000398307	0.021568992
Erc1	Q99MI1	ELKS/Rab6-interacting/CAST family member 1	0.00039715	0.006136254
Celsr2	Q9R0M0	Cadherin EGF LAG seven-pass G-type receptor 2	0.000369499	0.024618054
Rpl6	P47911	60S ribosomal protein L6	0.000357863	0.010527663
Olfm1	O88998	Olfactomedin 1	0.000356298	8.41787E-05
Tdrd6	P61407	Tudor domain-containing protein 6	0.000351949	0.027239048
Kcnb1	Q03717	Potassium voltage-gated channel subfamily B member 1	0.000346109	0.02338503
Atp1a1	Q8VDN2	Sodium/potassium-transporting ATPase subunit alpha-1	0.000324753	0.002380653
Rab11fip5	Q8R361	Rab11 family-interacting protein 5	0.000308667	0.042355884
Ccar1	Q8CH18	Cell division cycle and apoptosis regulator protein 1	0.0002935	0.028023158
Syt1	P46096	Synaptotagmin-1	0.000286519	0.001636541
Ap2a2	P17427	AP-2 complex subunit alpha-2	0.0002799	0.01849247
Ap2b1	Q9DBG3	AP-2 complex subunit beta	0.000255493	0.000142185
Syngap1	F6SEU4	Ras/Rap GTPase-activating protein SynGAP	0.00024615	0.016643339
Rpl7	P14148	60S ribosomal protein L7	0.000244439	0.000439493
Atp6v0a1	Q9Z1G4	V-type proton ATPase 116 kDa subunit a 1	0.000244369	3.38492E-05
Nptxr	Q99J85	Neuronal pentraxin receptor	0.000234577	0.001799195
Psd	Q5DTT2	PH and SEC7 domain-containing protein 1	0.000231972	0.037536052
Ptplad1	Q8K2C9	Very-long-chain (3R)-3-hydroxyacyl-CoA dehydratase 3	0.000222718	0.017326713
Baiap2	Q8BKX1	Brain-specific angiogenesis inhibitor 1-associated protein 2	0.000187	0.04648487
Gprin1	Q3UNH4	G protein-regulated inducer of neurite outgrowth 1	0.000169838	0.019971369
Tuba4a	P68368	Tubulin alpha-4A chain	0.000166288	0.001401173
Lsm11	Q8BUV6	U7 snRNA-associated Sm-like protein LSm11	0.000163591	0.00109894
Coro2b	Q8BH44	Coronin-2B	0.000157241	0.012456222
Mekk4	O08648	Mitogen-activated protein kinase kinase kinase 4	0.000157212	0.00360261
Aco2	Q99KI0	Aconitate hydratase, mitochondrial	0.000156051	0.02561227
Bcas1	Q80YN3	Breast carcinoma-amplified sequence 1 homolog	0.000151891	0.002311069
Grm7	Q68ED2	Metabotropic glutamate receptor 7	0.00014625	0.007328399
Pclo	Q9QYX7	Protein piccolo	0.000142753	0.027273825

Hspa8	P63017	Heat shock cognate 71 kDa protein	0.000140668	0.035675879
Srrm2	Q8BTI8	Serine/arginine repetitive matrix protein 2	0.000140522	0.044726099
Rims1	Q99NE5	Regulating synaptic membrane exocytosis protein 1	0.000117424	0.019844449
Iqca1l	A6H690	IQ and AAA domain-containing protein 1-like	0.00009528	0.021397764
Zfp786	Q8BV42	Krab domain-containing zinc finger protein	0.00004128	0.003431466
Gnaz	O70443	Guanine nucleotide-binding protein G(z) subunit alpha	0.000016318	0.008442232

B. α4-containing $GABA_A$Rs in WT

Protein	Uniprot ID	Description	Average SI_{GI}	p-value
Ttn	A2ASS6	Titin	0.003923373	0.046384038
Myo5a	Q99104	Unconventional myosin-Va	0.002567861	0.041552671
Dsp	E9Q557	Desmoplakin	0.001702051	0.027054259
Syne1	Q6ZWR6	Nesprin-1	0.001642225	0.043349018
Phrf1	A6H619	Phd and ring finger domain-containing protein 1	0.001583076	0.039587104
Vdac2	Q60930	Voltage-dependent anion-selective channel protein 2	0.001051761	0.010244726
Ankrd11	E9Q4F7	Ankyrin repeat domain-containing protein 11	0.001015605	0.009279662
Gli3	Q61602	Transcriptional activator GLI3	0.00098747	0.029294744
Vwa3b	A0A571BE33	Von Willebrand factor A domain containing 3B	0.000864469	0.002189713
Cacna1e	Q61290	Voltage-dependent R-type calcium channel subunit alpha-1E	0.000778112	0.025944887
Utp14a	Q640M1	U3 small nucleolar RNA-associated protein 14 homolog A	0.000583563	0.033980889
Snrnp200	Q6P4T2	U5 small nuclear ribonucleoprotein 200 kDa helicase	0.000561689	0.025248739
Atrx	Q61687	Transcriptional regulator ATRX	0.000538313	0.046143842
Mtr	A6H5Y3	Methionine synthase	0.000357104	0.015443802
Rad50	P70388	DNA repair protein RAD50	0.000351279	0.011657534
Ccdc62	E9PVD1	Coiled-coil domain-containing protein 62	0.000322904	0.025456012
Zc3h4	Q6ZPZ3	Zinc finger CCCH-type containing 4	0.00032281	0.040027915
Wdfy3	Q6VNB8	WD repeat and FYVE domain-containing protein 3	0.000228695	0.039984256
Huwe1	Q7TMY8	E3 ubiquitin-protein ligase HUWE1	0.000215969	0.035686929

C. α1-containing $GABA_A$Rs in S408/9A

Protein	Uniprot ID	Description	Average SI_{GI}	p-value
Sptan1	P16546	Spectrin alpha chain, non-erythrocytic 1	0.104947454	0.019724577
Sptbn1	Q62261	Spectrin beta chain, non-erythrocytic 1	0.043108863	0.021406288
Sptbn2	Q68FG2	Spectrin beta chain, non-erythrocytic 2	0.010669385	0.029664747
Myo5a	Q99104	Unconventional myosin-Va	0.009595686	0.00314924
Myh10	Q61879	Myosin-10	0.008850913	0.046369943
Cnp	P16330	2',3'-cyclic-nucleotide 3'-phosphodiesterase	0.00596544	0.041121581
Slc1a2	P43006	Excitatory amino acid transporter 2	0.004871453	0.036708636
Slc12a5	Q91V14	Solute carrier family 12 member 5	0.004279534	0.02834461
Srcin1	Q9QWI6	Src kinase signaling inhibitor 1	0.003511739	0.034182393
Gnao1	P18872	Guanine nucleotide-binding protein G(o) subunit alpha	0.003469868	0.04699541
Adamts20	P59511	A disintegrin-like and metallopeptidase with thrombospondin motifs 20	0.002675849	0.015190202
Slc4a10	Q5DTL9	Sodium-driven chloride bicarbonate exchanger	0.002125533	0.021176991
Atp9a	O70228	Probable phospholipid-transporting ATPase IIA	0.001641005	0.041560648
Akap5	D3YVF0	A-kinase anchor protein 5	0.001522048	0.011821443
Gabrg2	P22723	Gamma-aminobutyric acid receptor subunit gamma-2	0.00140266	0.027322348
Gphn	Q8BUV3	Gephyrin	0.000972962	0.035896407
Syt7	Q9R0N7	Synaptotagmin-7	0.000967148	0.029166072
Ank3	G5E8K5	Ankyrin-3	0.000827303	0.011794479
Dnajc13	D4AFX7	Dnaj heat shock protein family (Hsp40) member C13	0.000799165	0.041170398
Cntnap1	O54991	Contactin associated protein-like 1	0.000791669	0.039536828
Dmtn	Q9WV69	Dematin	0.000751791	0.038546747
Camkv	Q3UHL1	CaM kinase-like vesicle-associated protein	0.000731254	0.002272665
Cyfip2	Q5SQX6	Cytoplasmic FMR1-interacting protein 2	0.000662937	0.008816439
Tchh	A0A0B4J1F9	Trichohyalin	0.000640922	0.014785115
Zc3h13	E9Q784	Zinc finger CCCH domain-containing protein 13	0.000601407	0.017095605
Nrxn2	E9Q7X7	Neurexin ii	0.000586913	0.005483627
Spta1	P08032	Spectrin alpha chain, erythrocytic 1	0.000491936	0.022764077
Osbpl8	B9EJ86	Oxysterol-binding protein-related protein 8	0.000463157	0.000933063
Atp10a	O54827	Probable phospholipid-transporting ATPase VA	0.000461848	0.008887311

Ankrd26	Q811D2	Ankyrin repeat domain 26	0.000457443	0.026052131
Xrn2	Q9DBR1	5'-3' exoribonuclease 2	0.00043685	0.017032608
Nckap1	P28660	Nck-associated protein 1	0.000393258	0.015002766
Ap2a1	P17426	AP-2 complex subunit alpha-1	0.000376401	0.009568735
Ablim2	Q8BL65	Actin-binding LIM protein 2	0.000340787	0.047572458
Adcy9	P51830	Adenylate cyclase type 9	0.000306614	0.028321727
Eif4g3	Q80XI3	Eukaryotic translation initiation factor 4 gamma 3	0.000223623	0.037478494
Mdn1	A2ANY6	Midasin	0.000145033	0.00206862
Arl6ip4	Q9JM93	ADP-ribosylation factor-like protein 6-interacting protein 4	8.07104E-05	0.014267835

D. α4-containing $GABA_A$Rs in S408/9A

Protein	Uniprot ID	Description	Average SI_{GI}	p-value
Loxl4	Q924C6	Lysyl oxidase homolog 4	0.002173241	0.031018965
Adamts15	P59384	A disintegrin-like and metallopeptidase with thrombospondin motifs 15	0.001415625	0.000740337
Nod2	Q8K3Z0	Nucleotide-binding oligomerization domain-containing protein 2	0.001222888	0.042343995
Slk	O54988	STE20-like serine/threonine-protein kinase	0.000417069	0.048886269
Tchh	A0A0B4J1F9	Trichohyalin	0.000411174	0.001866315
Rin3	P59729	Ras and Rab interactor 3	0.000290562	0.034873024
Rps26	P62855	40S ribosomal protein S26	0.000255303	0.006955658
Gm7072	A0A3B2WCF4	Predicted gene 7072	0.000188321	0.019538646
Agbl3	Q8CDP0	Cytosolic carboxypeptidase 3	0.000160073	0.042485277
