# Supplementary figures and images for: Analyzing the mechanisms that facilitate the subtype-specific assembly of γ-aminobutyric acid type A receptors
Source: Front Mol Neurosci. 2022 Oct 3;15:1017404. doi: 10.3389/fnmol.2022.1017404 (PMC9574402; doi:10.3389/fnmol.2022.1017404)

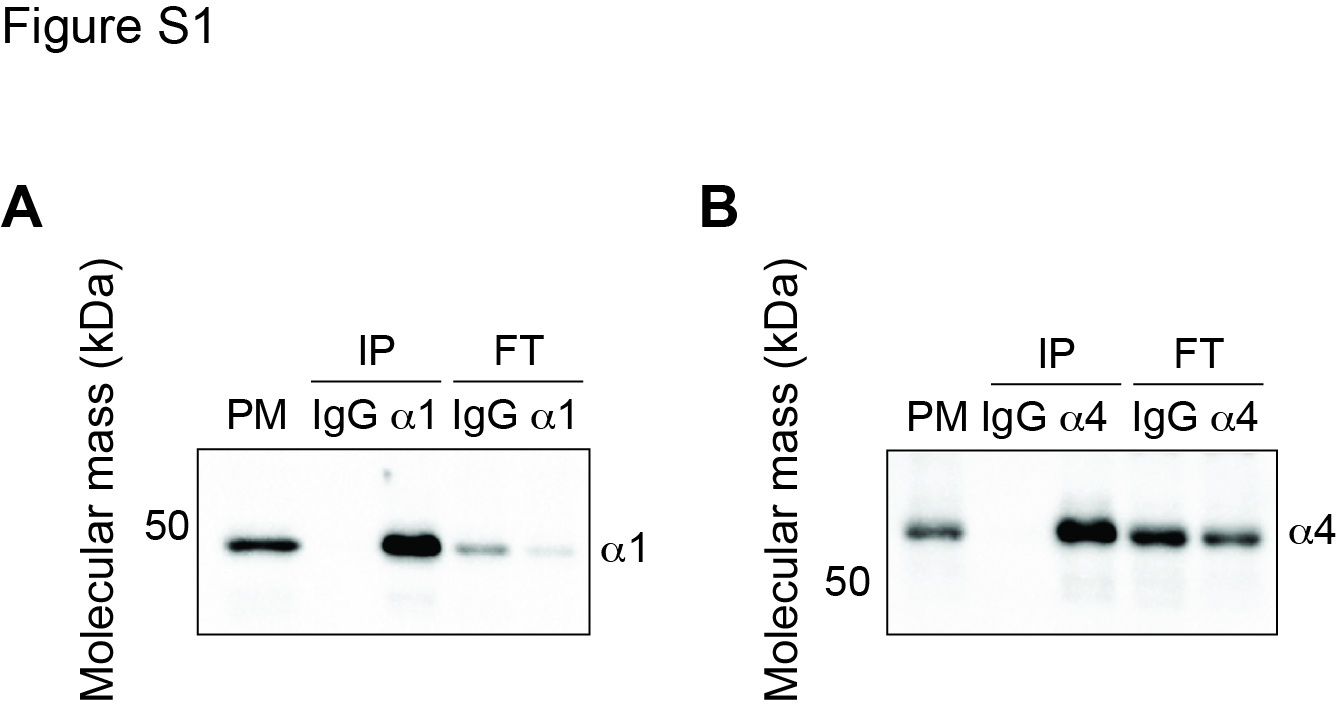

Supplement: Supplementary file 4 [file Image_1.JPEG]

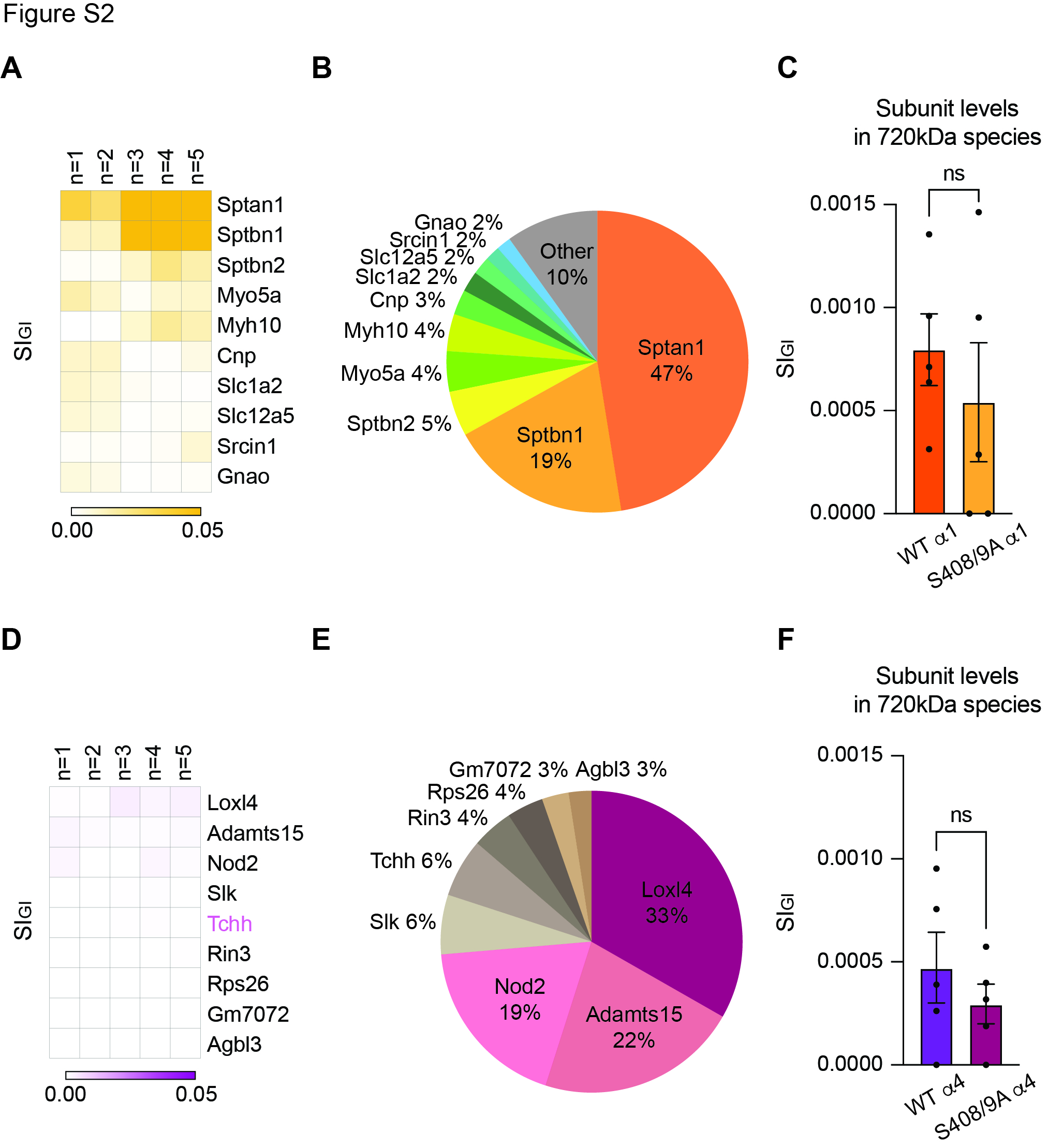

Supplement: Supplementary file 5 [file Image_2.JPEG]

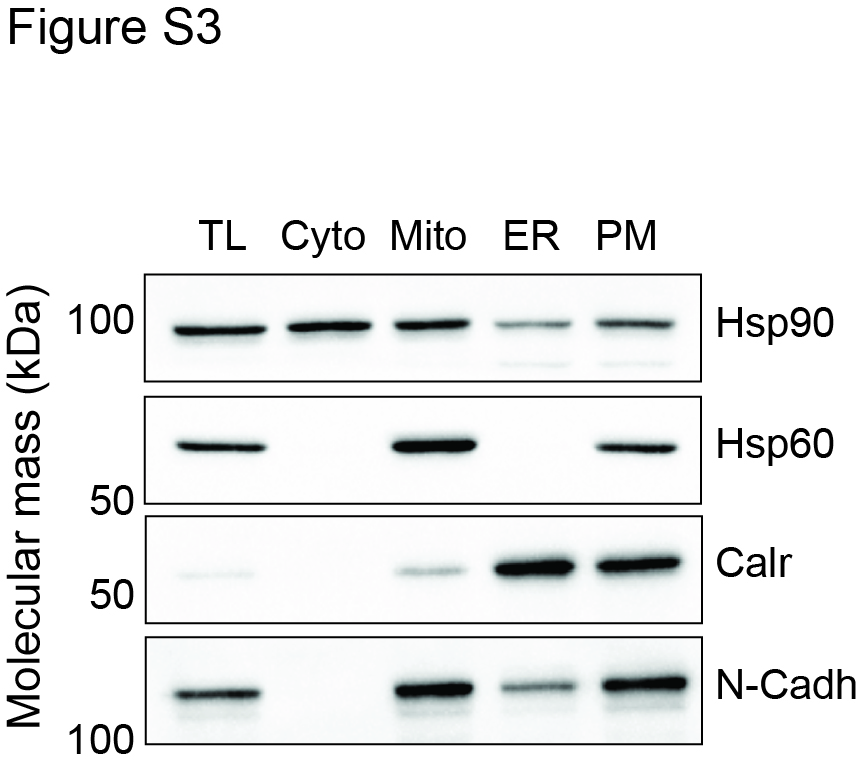

Supplement: Supplementary file 6 [file Image_3.JPEG]
